# Nitrogen Sources Reshape Microbial Community Assembly and Interaction Networks to Regulate Straw Decomposition and Carbon Release in Cropland Soils of the North China Plain

**DOI:** 10.3390/microorganisms14081753

**Published:** 2026-08-09

**Authors:** Yaxian Wang, Tengfei Guo, Chao Ai, Shuyan Fan, Yilun Wang, Fengjun Zheng, Qian Zhang

**Affiliations:** 1 State Key Laboratory of High-Efficiency Production of Wheat-Maize Double Cropping, College of Resources and Environment, Henan Agricultural University, Zhengzhou 450046, China; wangyaxian1101@163.com (Y.W.); fanshuyan0807@163.com (S.F.); wangyilunrl@henau.edu.cn (Y.W.); zfengjun@henau.edu.cn (F.Z.); 2 College of Agronomy, Henan Agricultural University, Zhengzhou 450046, China; 3 Institution of Plant Nutrition and Environmental Resources, Henan Academy of Agricultural Sciences, Zhengzhou 450002, China; guotengfei@hnagri.org.cn; 4 Institute of Agricultural Resources and Regional Planning, Chinese Academy of Agricultural Sciences, Beijing 100081, China; aichao@caas.cn

**Keywords:** straw decomposition, nitrogen forms, microbial community assembly, microbial interaction networks, carbon cycling, agricultural soils

## Abstract

The decomposition of crop residue is indispensable for carbon (C) cycling in agricultural ecosystems, and nitrogen (N) application has a pronounced effect on this by regulating the C/N ratio and microbial growth strategy. This study investigated how different inorganic and organic nitrogen sources interact with soil properties to regulate straw decomposition, nutrient release, microbial community assembly, and microbial interaction networks across typical cropland soils of the North China Plain. Five treatments, i.e., no N addition, urea, calcium nitrate, yeast derivatives, and glutamate, were conducted in fluvo-aquic soil, lime concretion black soil, cinnamon soil, and yellow-cinnamon soil. We found that soil type strongly regulated straw decomposition dynamics, with cumulative decomposition rates varying greatly among the four soil types after 180 days, ranging from 55.78% to 68.75%. In particular, the lime concretion black soil exhibited the highest straw decomposition and C release rates, which harbored higher relative abundances of potential straw-decomposing taxa, including *Luteibacter*, *Chitinophaga*, *Dothideomycetes*, and *Leotiomycetes*. Bacterial community assembly shifted from stochastic dominance at early stages to deterministic selection at later stages, whereas fungal community assembly remained predominantly stochastic with increasing dispersal limitation during late decomposition. The dominant bacterial phyla were Proteobacteria and Bacteroidota, and the fungal community composition succeeded from *Alternaria*, *Rhizopus*, *Cladosporium* and *Meyerozyma* to *Chaetomium*, *Schizophecium* and *Apodus*. Compared with no N addition, yeast derivatives significantly increased the straw decomposition rate in fluvo-aquic soil, lime concretion black soil, and cinnamon soil by 15.00, 10.66, and 12.19 percentage points, respectively, within 14 days. This is related to the enhanced complexity of the bacterial co-occurrence network, among which *Altererythrobacter*, *Sphingomonas*, *Candidatus_Xiphinematobacter*, *Devosia*, and *Chitinophaga* were identified as key microbial groups. Glutamate stimulated later decomposition by modifying the fungal community structure and strengthening fungal interactions centered on *Alternaria* and *Schizothechium*. These findings reveal how N forms regulate straw decomposition through soil-dependent microbial assembly and interaction networks, providing a mechanistic basis for optimizing nitrogen management strategies for efficient straw nutrient recycling.

## 1. Introduction

The global soil carbon (C) pool is 3–4 times larger than the vegetation C pool [1], with crop residue incorporation being a key contributor, particularly in agroecosystems [2,3]. Annually, 4.5 billion tons of crop residue are produced worldwide, with 840 million tons in China, 47% of which is directly returned to the soil [4]. Numerous studies highlight the benefits of crop residue return, including sustaining crop yield, increasing soil organic C storage, improving soil fertility and structure, and stimulating soil microbial diversity [5,6,7]. Although combining straw return with mineral fertilization can increase soil organic carbon (SOC) by 0.12–0.16 Mg C yr^−1^, the efficiency of SOC stock improvement in response to crop residue incorporation is still less than 30% [8]. Therefore, the specific regulatory mechanisms governing efficient straw return remain to be elucidated.

Nitrogen (N) fertilizer is a crucial approach to enhance crop production by 30–50%, and support over half of the global population [9]. Extensive research has investigated the mechanisms of N addition regulating the decomposition and transformation process of crop residue [10,11,12,13,14,15], finding that (1) the application of N fertilizer accelerates residue decomposition by enhancing the stoichiometric properties of residue substrate in the early stage and favoring the activity of copiotrophic microorganisms; (2) elevated N availability significantly inhibits the extracellular enzyme activities of microorganisms, reducing microbial ability for C source acquisition and therefore improving its C utilization efficiency; (3) N enrichment alters the cooperative and competitive dynamics within microbial populations, thereby affecting the composition of straw-decomposing microbial communities; and finally, (4) N enrichment can decrease the rate and degree of crop residue decomposition and increase the oxidation and stability of lignin-like compounds and promote their accumulation. However, limited research has focused on how microbial preferences for exogenous N types influence the decomposition and transformation of crop residue.

At present, most studies have reported the role of inorganic N sources in straw decomposition. For example, Wang et al. [16] reported that the addition of ammonium nitrate enhanced the efficiency of microbial N use in the decomposition of maize straw, thereby inhibiting the mineralization of native soil organic matter. Compared with inorganic N, organic N compounds dominate the labile pools in N-limited environments [17], and microorganisms may extensively rely on organic N monomers, such as amino acids, to meet their N requirements [18], thereby promoting organic matter decomposition. The application of poly-γ-glutamic acid (γ-PGA) was found to improve potassium bioavailability and benefit plants by driving cross-feeding interaction between rhizosphere bacteria [19]. The preferential uptake of acidic amino acids such as glutamic acid by amino acid transporters enables them to rapidly enter the microbial metabolic network, which ultimately results in performance advantages in straw decomposition through restructuring the microbial community and enriching core functional groups [20,21]. Yeast metabolites, which are downstream processed products of nutritional yeast, rich in easily absorbed nutrients from aerobic fermentation, have shown potential to promote plant growth as a novel organic N source [22]. In fact, crop straw residue possesses a large specific surface area and typically carries negative surface charges, implying that different N forms, owing to their distinct ionic properties, may exhibit markedly different adsorption/ion exchange behaviors on the residue surface [23]. More importantly, straw decomposition is not a homogeneous process; the surface physicochemical properties evolve continuously over time [24], and the dynamic adsorption and release of N directly alter the N availability of microbial decomposers at different decomposition stages, thereby filtering for microbial functional groups with specific life-history strategies. For instance, most bacterial communities are typical r-strategists that readily utilize labile substrates in straw, whereas fungal taxa, equipped with more sophisticated extracellular enzymatic systems, possess stronger capabilities to degrade recalcitrant components such as lignin and generally exhibit better adaptability to low-N environments [10,25,26]. Nevertheless, further studies are still required to disentangle the mechanisms by which different N sources (e.g., oxidized vs. reduced N forms, and chemical vs. organic N forms) regulate microbially driven crop residue decomposition to identify the optimal N source for straw incorporation.

Essential microbial taxa responsible for soil nutrient transformation, such as crop residue decomposers, primarily originate from the adjacent soil microflora [27,28]. Consequently, residue decomposition also highly depends on soil properties, with climate conditions and physicochemical characteristics along biogeographical distance under various soil types associated with strong soil-specific responses of decomposer communities [8,29,30]. Finn et al. [31] found that clay texture soil facilitates faster microbial colonization on crop residue, leading to accelerated wheat straw mineralization and enhanced soil mineral-bound C pools compared with sandy loam soil [32]. Fluvo-aquic soil, cinnamon soil, lime concretion black soil, and yellow-cinnamon soil are predominant arable soils of the North China Plain of China. The heterogeneous characteristics of these soils might create varying niches that support microbial activity essential for the decomposition of crop residue. Therefore, in this study, a 180-day nylon mesh bag decomposition experiment was conducted across four soil types under a wheat–maize rotation system to examine the decomposition of maize residue with the addition of various N sources during the wheat growth period. Decomposition rates, C and N release, organic component decomposition, and microbial community diversity were determined to test the following hypotheses: (1) soil characteristics shape unique ecological clusters in different soil types; (2) N source addition, particularly an organic source, accelerates maize residue decomposition due to enhanced nutrient availability; and (3) residue-decomposing microbial community composition shows clear succession over decomposition and accelerates the growth of specific microbial taxa responding to the N source. The findings of this study aim to provide an important reference for optimizing strategies for crop residue utilization in agricultural soils.

## 2. Materials and Methods

### 2.1. Study Site and Experimental Design

Eleven sites representing four typical soil types (fluvo-aquic soil, cinnamon soil, lime concretion black soil, and yellow-cinnamon soil) were chosen in the North China Plain. According to the FAO-UNESCO soil classification system, these soils are classified as Fluvisols, Cambisols, Vertisols and Luvisols. The fundamental climatic and physicochemical soil characteristics are listed in Table S1.

Maize residue was collected during the harvest period, dried at 60 °C, and then cut into 1–2 cm pieces. The C and N contents of the maize residue were determined to be 42.31% and 0.46%, respectively. Five fertilizer treatments were implemented using the nylon mesh bag method, with four replicates per treatment. Each nylon bag (10 cm × 15 cm, 62 μm mesh size) was filled with 14 g of maize residue (corresponding to a full dose of returned maize residue at 8000 kg/hm^2^ in the field) and supplemented with one of the following N sources: no N fertilizer addition (T1), urea (T2), calcium nitrate (T3), yeast derivatives (T4), or glutamate (T5). The addition amount of each N source was calculated to achieve a uniform initial C/N ratio of 25:1 for the total mixture inside each bag. According to previous reports, this ratio is considered an optimal threshold for promoting straw decomposition and balancing microbial energy and nutrient requirements while avoiding microbial nitrogen limitation [33,34]. The detailed addition amounts for each treatment are provided in Table 1. The bags, buried horizontally at a depth of 15 cm, were retrieved and transported to the laboratory under low temperature on days 7, 14, 30, 90, and 180 post-burial of maize residue during the winter wheat season from October 2022 to June 2023.

### 2.2. Determination of Residue Nutrient Release and Organic Component Decomposition Rates

After the removal of soil and roots adhering to the bag’s surface, the residue was completely retrieved and transferred into a clean self-sealing bag. A portion of the maize residue was dried at 65 °C to determine its mass and calculate the decomposition rate under different N fertilization treatments. The C and N contents of the straw residue were determined using an elemental analyzer (Elementar Analysen Systeme GmbH, Hanau, Germany). The organic components of the maize residue were determined using a cellulose, hemicellulose, and lignin content kit (Suzhou Grace Biotechnology Co., Ltd., Suzhou, China).

### 2.3. DNA Sequencing

Subsequently, a subset of samples across four sampling sites from all remaining straw residues was selected for DNA extraction and bioinformatics analysis, with each site uniquely representing one of the four soil types investigated in this study. Total microbial DNA associated with the residue was extracted using FastDNA SPIN Kit (MP Biomedicals, Santa Ana, CA, USA) according to the manufacturer’s instructions. The concentration and quality of DNA were assessed via agarose gel electrophoresis and a Nanodrop spectrophotometer (Nanodrop, Peq Lab, Erlangen, Germany). Both the V3-V4 region of the bacterial 16S rRNA gene and the fungal ITS2 region of each sample were amplified in duplicate on an ABI GeneAmp 9700 PCR system, and the PCR amplification products were purified using an AxyPrep PCR Clean-up kit (Axygen Biosciences, Union City, CA, USA). The bacterial primers were 338F (5′-ACTCCTACGGGAGGCAGCA-3′) and 806R (5′-GGACTACHVGGGTWTCTAA-3′). The fungal primers were ITS3F (5′-GCATCGATGAAGAACGCAGC-3′) and ITS4R (5′-TCCTCCGCTTATTGATATGC-3′). Equimolar PCR products ligated with barcode oligonucleotides were paired-end-sequenced on the Illumina MiSeq PE300 platform (Majorbio Co., Ltd., Shanghai, China). The obtained sequences were filtered and spliced using Trimmomatic and FLASH. Finally, 12421246 high-quality bacterial sequences and 14990754 fungal sequences were obtained from 183 samples. The USEARCH and UPARSE algorithms (version 7.1 http://drive5.com/uparse/, accessed on 22 July 2025) were used to cluster the sequences into the operational taxonomic units (OTUs) at a 97% similarity level and remove the chimeras to obtain the representative sequences of OTUs. The representative sequences of the 16S rRNA gene and ITS gene were taxonomically compared with the Silva database (Release 138 https://www.arb-silva.de/, accessed on 22 July 2025) and the UNITE database (Release 8.0 https://unite.ut.ee/, accessed on 22 July 2025), respectively, using RDP Multi-Classifier (https://github.com/rdpstaff/classifier, accessed on 22 July 2025) at a threshold of 0.7. The sequencing data are available in the NCBI Sequence Read Archive (www.ncbi.nlm.nih.gov/, accessed on 22 July 2025) with accession numbers PRJNA1249589 and PRJNA1249631. To reduce the impact of sequencing depth on subsequent analysis, the minimum sequence number for each sample was randomly selected and 2498 bacterial OTUs and 776 fungal OTUs were obtained for further analyses.

### 2.4. Statistics Analysis

The multiple comparisons of maize residue decomposition rate, C and N release, and organic components in different soils under N fertilizations along different decomposition time were tested with Ducan’s test at a significance level of *p* < 0.05 using IBM SPSS 26.0 software. The alpha diversity and community composition of maize residue-decomposing bacterial and fungal communities were calculated and the Kruskal–Wallis *H* test was carried out on the community succession along with decomposition time in four soil types on the Majorbio Cloud platform (https://www.majorbio.com/). Principal coordinate analysis (PCoA), ANOSIM analysis, and permutational multivariate analysis of variance (PERMANOVA) were carried out based on the Bray–Curtis distance matrix using the “ade4” (version 1.7-22) and “vegan” (version 2.6-8) packages in R v4.3.3 software. The correlation between microbial community structure and environmental variables was determined by the Mantel test with the “vegan” (version 2.6-8) package.

Distance decay relationship (DDR) analysis was conducted based on the “geosphere” (version 1.6-8) and “vegan” (version 2.6-8) packages to analyze the similarity variation of straw-decomposing bacterial and fungal communities along with geographical distance. Null model analysis was performed to quantify the ecological processes of bacterial and fungal assemblages according to Zhou et al. [35]. The variations in both phylogenetic diversity and taxonomic β-diversity metrics, namely the β-nearest taxon index (βNTI) and Bray–Curtis-based Raup–Crick (RC_Bray_), were calculated using null model analysis (999 randomizations) to quantify the contribution of deterministic and stochastic processes. Briefly, |βNTI| > 2 indicated the dominance of deterministic dominant processes (variable (βNTI > 2) or homogeneous selection (βNTI < −2)). |βNTI| < 2 signified stochastic dominated processes; in this case, RC_Bray_ > 0.95 indicated dispersal limitation, RC_Bray_ < −0.95 indicated homogenizing dispersal, and |RC_Bray_| < 0.95 indicated undetermined processes, mostly attributable to weak dispersal and selection, diversification, and drift [36].

The “psych” (version 2.4.6.26) package was employed to create a microbial co-occurrence network derived from the Spearman correlation matrix. Correlation coefficients ρ > 0.8 and FDR-corrected *p* < 0.001 were set to improve the network robustness with the “multtest” (version 2.58.0) package in R v4.3.3 [37]. A set of topological properties, including node and edge numbers, positive and negative edges, bacterial and fungal interactions, edge density, and clustering coefficient, were calculated with the “igraph” (version 2.1.1) package in R v4.3.3 [38]. The visualization of this network was accomplished using Gephi v4.10.1 (http://gephi.github.io/, accessed on 20 September 2025), a powerful tool for network visualization and analysis in complex systems. Moreover, the within-module degree (z-score) and among-module degree (c-score) of nodes were calculated, and the nodes defined as kinless hubs (z-score > 2.5; c-score > 0.6), module hubs (z-score > 2.5; c-score < 0.6), or connectors (z-score < 2.5; c-score > 0.6) were identified as key taxa [39].

To further link microbial community characteristics to straw decomposition performance, we used as predictive factors the straw decomposition rate at D180 as the response variable, the alpha-diversity indices (Shannon and Sobs of bacteria and fungi), the beta-diversity represented by the first two principal coordinate axes (PC1 and PC2) derived from PCoA based on the Bray–Curtis distance, and the relative abundance of OTUs identified as key nodes in each treatment co-occurrence network. The random forest model was conducted using the “randomForest” (version 4.7-1.2) package in R v4.3.3 software for two types of organic N source treatments.

## 3. Results

### 3.1. Decomposition Patterns of Maize Residue in Typical Soils Under Incorporation of Different N Sources 

**Figure 1:**
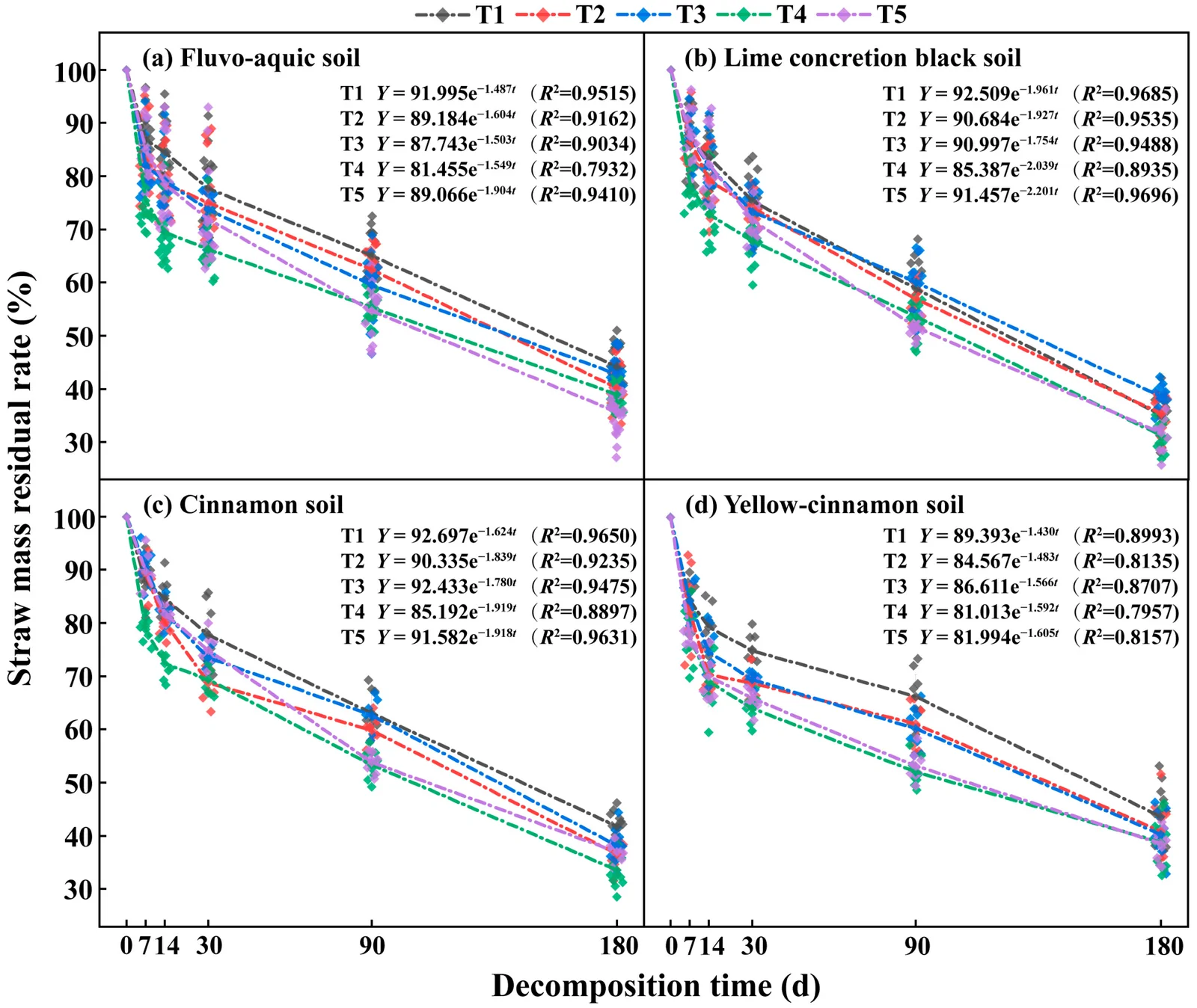
Straw decomposition rate under various nitrogen source treatments in different soil types. *Y*—straw mass residual rate (%); t—decomposition time (yr); e—natural constant; *R*^2^—the goodness-of-fit of the equation.

The decomposition of maize residue initially proceeded rapidly, and then slowed in later stages (Figure 1). Lime concretion black soil exhibited the highest decomposition rate, followed by cinnamon soil. By D180, the cumulative decomposition rates of maize residue on fluvo-aquic soil, lime concretion black soil, cinnamon soil, and yellow-cinnamon soil reached 55.78–64.35%, 61.42–68.75%, 58.28–66.44%, and 56.30–61.30%, respectively. T4 significantly enhanced residue decomposition on fluvo-aquic soil, lime concretion black soil, and cinnamon soil up to D30, while T5 accelerated residue decomposition in the middle and later periods. Both T4 and T5 treatments strongly promoted residue decomposition in yellow-cinnamon soil.

The residue C release rate mirrored the residue decomposition rate, initially rapid and then gradually slowing down, which was fastest in lime concretion black soil (Figure S1). The C release rates were 55.69–64.95%, 63.90–69.76%, 58.71–66.88%, and 56.91–61.50% for fluvo-aquic soil, lime concretion black soil, cinnamon soil, and yellow-cinnamon soil at D180, respectively. T4 significantly enhanced early C release in fluvo-aquic soil, lime concretion black soil, and cinnamon soil, while T5 accelerated C release after D30. Both T4 and T5 treatments strongly promoted C release in yellow-cinnamon soil. Compared with T1, the addition of various N sources enhanced residue N release, with T4 showing superior performance before D30 (Figure S2). In the first 14 days, the N release rate in fluvo-aquic, lime concretion black, and cinnamon soils was notably lower under T5 treatment compared to other exogenous N treatments, but it accelerated after day 14 (D14). N enrichment was observed in all exogenous N treatments at D180.

Straw cellulose and hemicellulose showed higher decomposition rates than lignin in different soils (Figure S3). The decomposition of cellulose, hemicellulose, and lignin in lime concretion black soil and cinnamon soil was relatively fast. At D180, the decomposition rates of cellulose for fluvo-aquic soil, lime concretion black soil, cinnamon soil, and yellow-cinnamon soil were 57.79–69.84%, 65.79–72.11%, 67.99–77.04%, and 49.37–55.99%, respectively; the decomposition rates of hemicellulose were 52.47–61.63%, 56.15–63.95%, 63.03–71.37%, and 41.85–54.10%, respectively; and the decomposition rates of lignin were 21.99–30.90%, 37.54–53.22%, 32.64–46.04%, and 30.23–36.94%, respectively. In particular, compared with other exogenous N treatments, the decomposition rates of straw cellulose, hemicellulose, and lignin were higher under T4 and T5 treatments. The straw decomposition-promoting effect of the T4 treatment was particularly significant during the first 14 days of decomposition, while the decomposition rate rapidly increased under the T5 treatment in the later stage.

### 3.2. Alpha-Diversity of Residue-Decomposing Bacterial and Fungal Communities Under Different N Source Types

**Figure 2:**
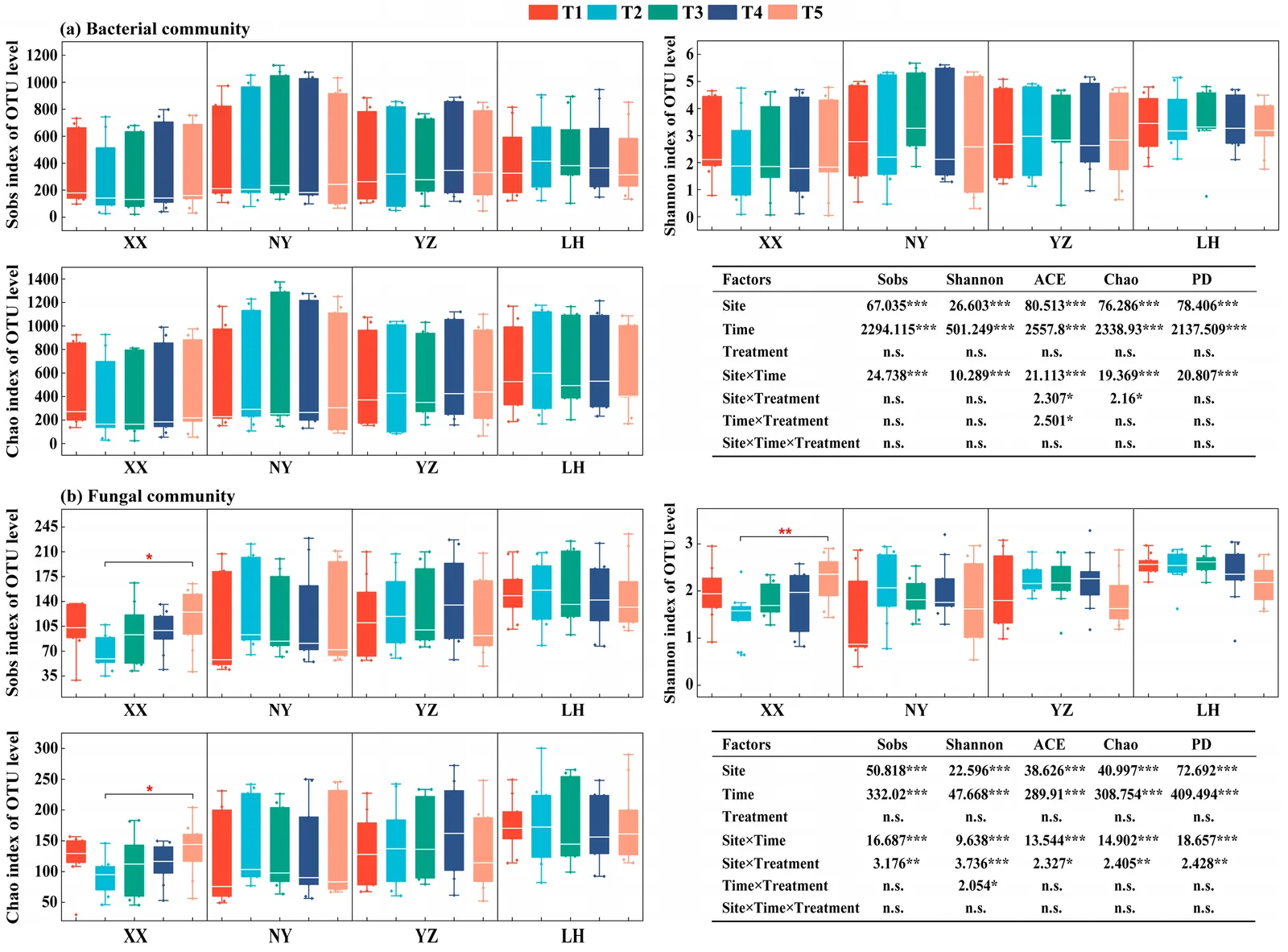
The α-diversity of straw-decomposing bacterial (**a**) and fungal (**b**) communities under various nitrogen source treatments in different soil types. * *p* < 0.05, ** *p* < 0.01, *** *p* < 0.001, n.s.—no significant differences. Both red and black asterisks represent the same significance levels.

**Figure 3:**
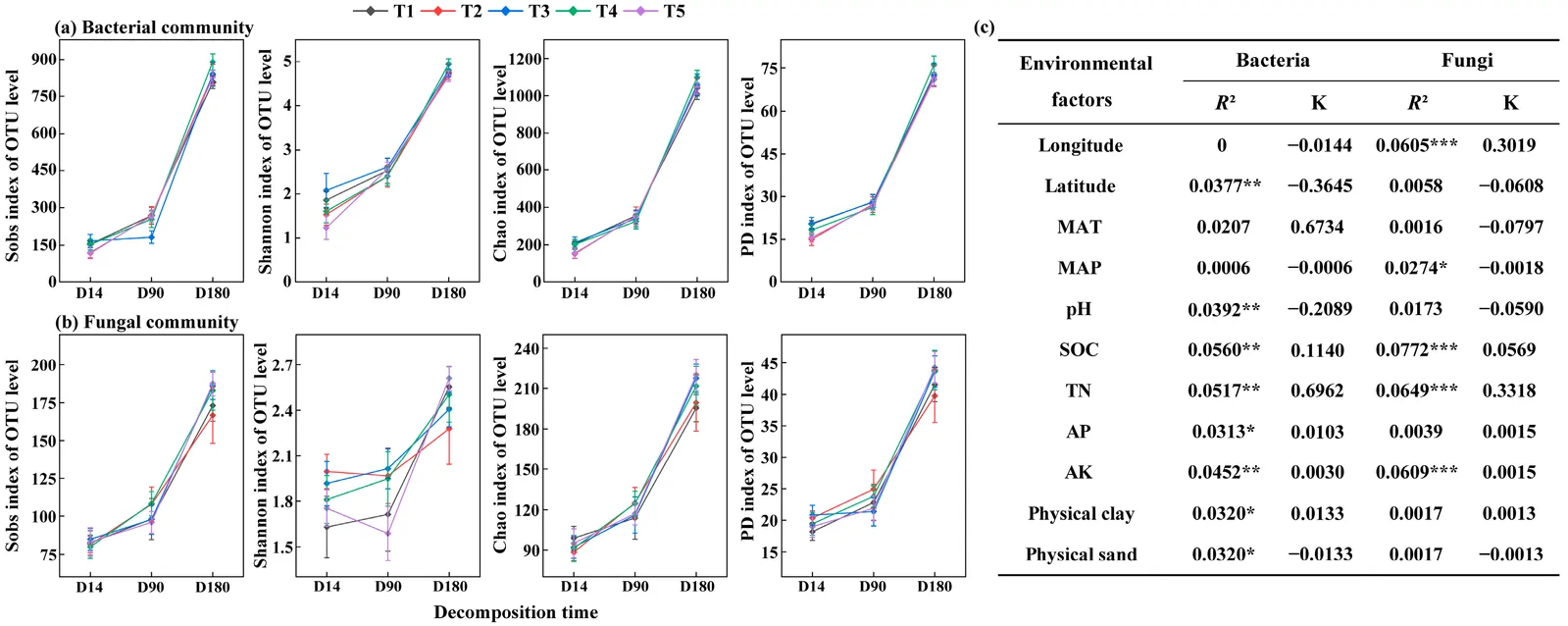
Changes in α-diversity of straw-decomposing bacterial (**a**) and fungal (**b**) communities over time and their linear regression analysis with environmental factors (**c**). * *p* < 0.05, ** *p* < 0.01, *** *p* < 0.001, n.s.—no significant differences; MAT—mean annual temperature; MAP—mean annual precipitation; SOC—soil organic carbon; TN—total nitrogen; AP—available phosphorus; AK—available potassium; *R*^2^—the coefficient of determination; K—the slope of the linear regression equation.

Three-way ANOVA revealed that soil type, decomposition duration, and their interaction significantly influenced the alpha-diversity of residue-decomposing bacterial and fungal communities, with the highest alpha-diversity index observed at D180 (Figure 2 and Figure 3a,b). Bacterial community alpha-diversity exhibited a significant positive correlation with SOC, TN, AP, AK, and soil physical clay content, and a significant negative correlation with latitude, pH, and soil physical sand content (Figure 3c), with higher diversity observed in yellow-cinnamon soil and lower in fluvo-aquic soil (Figure 2a). Additionally, longitude, SOC, TN, and AK had a significant and strong positive effect on the alpha-diversity of the fungal decomposing community (Figure 3c). Notably, the alpha-diversity of residue-decomposing fungal communities was also significantly affected by the interaction between soil type and exogenous N treatment, and the alpha-diversity of fungal communities showed a significant difference between no N fertilization and inorganic N application (T1–T3), which was higher in yellow-cinnamon soil. Compared with T2, T5 significantly improved the Sobs, Chao, and Shannon indices of residue-decomposing fungi in fluvo-aquic soil (Figure 2b).

### 3.3. The Community Structure of Residue-Decomposing Bacteria and Fungi Under Different N Source Types

**Figure 4:**
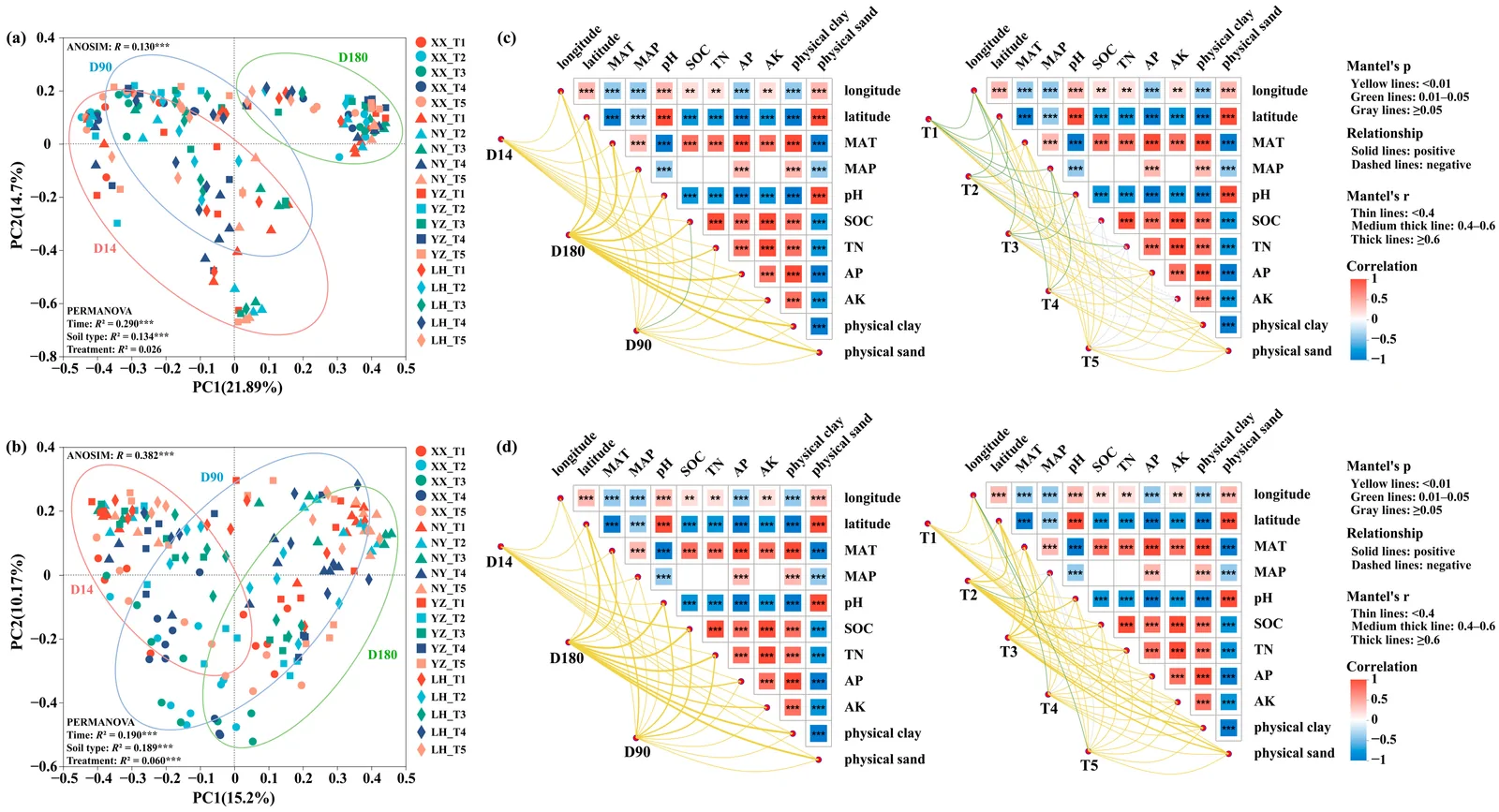
PCoA (**a**,**b**) and Mantel’s test analysis (**c**,**d**) of straw-decomposing bacterial and fungal communities. ** *p* < 0.01, *** *p* < 0.001. MAT—mean annual temperature; MAP—mean annual precipitation; SOC—soil organic carbon; TN—total nitrogen; AP—available phosphorus; AK—available potassium.

The community structure of residue-decomposing bacteria and fungi varied significantly across different decomposition stages (Figure 4a,b). A notable correlation existed between the decomposing microbial community structure and environmental factors during residue decomposition, which was especially stronger at D180 (Figure 4c,d). While different N sources did not significantly alter the bacterial community structure, they significantly impacted the fungal community structure (Figure 4a,b). Bacterial communities across different N treatments showed significant positive correlations with climate, pH, AP, and soil texture. Fungal communities in the T1, T4, and T5 treatments were significantly influenced by soil fertility, whereas in the T2 and T3 treatments they were strongly correlated with latitude and soil texture (Figure 4c,d). Over time, the similarity of residue-decomposing bacterial and fungal community structures significantly decreased with geographical distance (Figure S4). At D14 and D90, bacterial communities exhibited greater changes than fungal communities, whereas the opposite pattern was observed at D180 (Figure S4a). The residue-decomposing bacterial community structures were more similar than those of the fungal community among different N fertilizer treatments. Compared to other treatments, the bacterial and fungal communities under T5 treatment showed less variation with geographical distance (Figure S4b).

**Figure 5:**
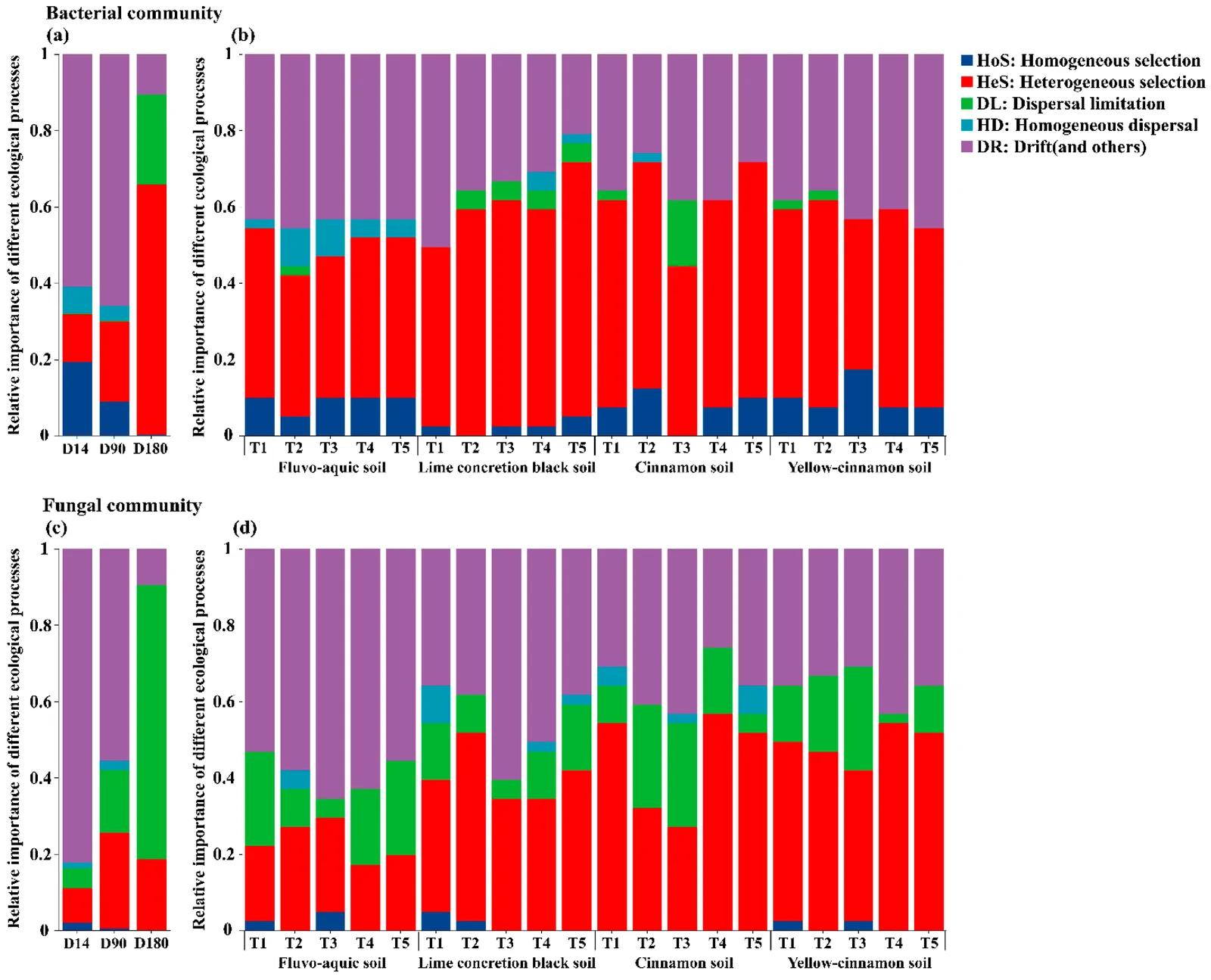
The community construction process of straw-decomposing bacterial (**a**,**b**) and fungal (**c**,**d**) communities.

Null model analysis further revealed that deterministic processes increasingly favored the assembly of residue-decomposing bacterial community over time, with heterogeneous selection prevailing at D180 (Figure 5a). The shift of bacterial community structure on lime concretion black soil and cinnamon soil was primarily driven by deterministic processes (heterogeneous selection) (Figure 5b), while fungal community assembly was predominantly shaped by stochastic processes, with dispersal limitation becoming notably more pronounced at D180 (Figure 5c). In contrast, stochastic processes had a substantial impact on the fungal community structure in fluvo-aquic soil (Figure 5d).

### 3.4. The Community Composition of Residue-Decomposing Bacteria and Fungi Under Different N Source Types

**Figure 6:**
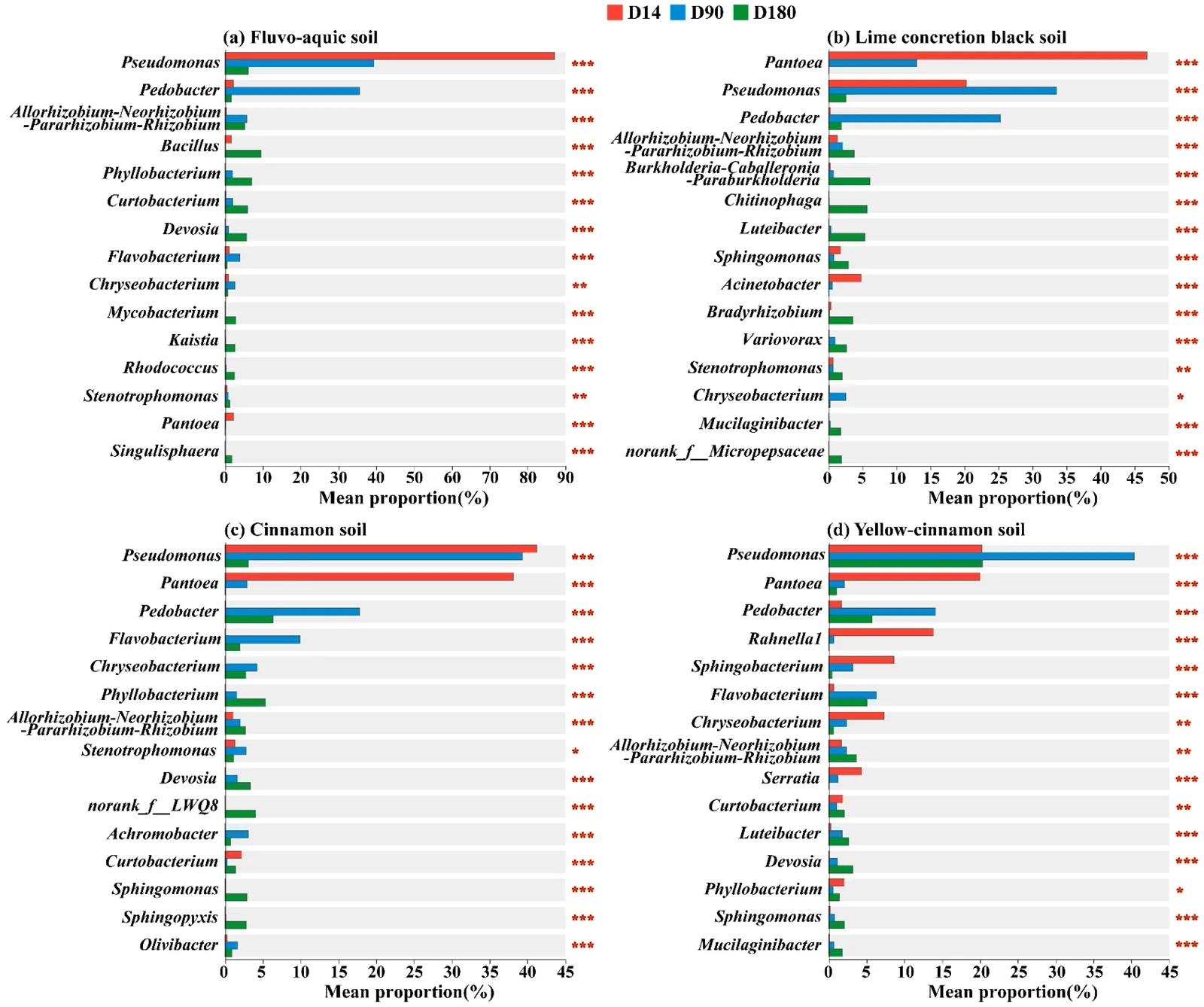
Kruskal–Wallis *H* test of straw-decomposing bacterial community at the genus level. * *p* < 0.05, ** *p* < 0.01, *** *p* < 0.001.

**Figure 7:**
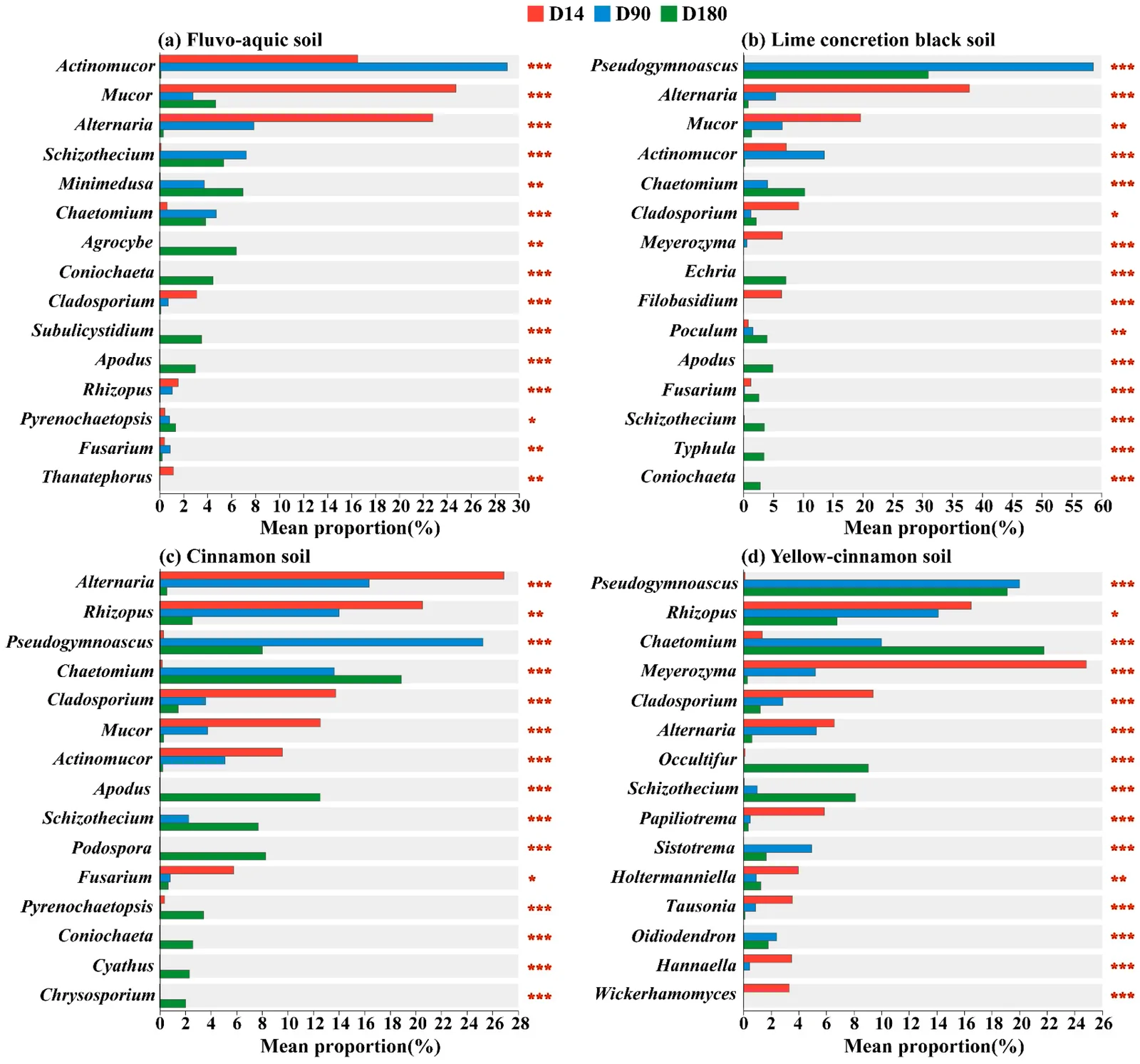
Kruskal–Wallis *H* test of straw-decomposing fungal community at the genus level. * *p* < 0.05, ** *p* < 0.01, *** *p* < 0.001.

The residue-decomposing bacteria in response to various N sources across distinct soil types were predominantly represented by Proteobacteria and Bacteroidota (Figure S5a). Notably, Sordariomycetes and Mucoromycetes exhibited increased relative abundance under T2–T5 treatments in fluvo-aquic soil and cinnamon soil, whereas Dothideomycetes and Leotiomycetes were enriched in lime concretion black soil (except T4) (Figure S5b). Over time, there were significant shifts in the bacterial and fungal community composition during the decomposition process. By D180, there was a notable rise in the relative abundance of *Bacillus*, *Phyllobacterium*, *Curtobacterium*, and *Devosia* in fluvo-aquic soil; *Burkholderia-Caballeronia-Paraburkholderia*, *Chitinophaga*, *Luteibacter*, and *Bradyrhizobium* in lime concretion black soil; *Phyllobacterium* and *Sphingomonas* in cinnamon soil; and *Devosia* in yellow-cinnamon soil (Figure 6). Furthermore, certain fungi such as *Agrocybe*, *Coniochaeta*, *Subulicystidium*, *Apodus*, *Echria*, *Typhula*, *Podospora*, *Pyrenochaetopsis*, *Coniochaeta*, *Cyathus*, *Chrysosporium*, and *Occultifur* displayed significantly higher abundance during this period (Figure 7). Interestingly, some microbial communities involved in maize residue decomposition exhibited similar changes in relative abundance across different soil types, including bacteria such as *Pseudomonas*, *Pedobacter*, and *Pantoea*, and fungi such as *Alternaria*, *Schizothecium*, *Chaetomium*, and *Cladosporium* (Figure 6 and Figure 7).

### 3.5. Co-Occurrence Networks of Residue-Decomposing Bacteria and Fungi Under Different N Source Types

**Figure 8:**
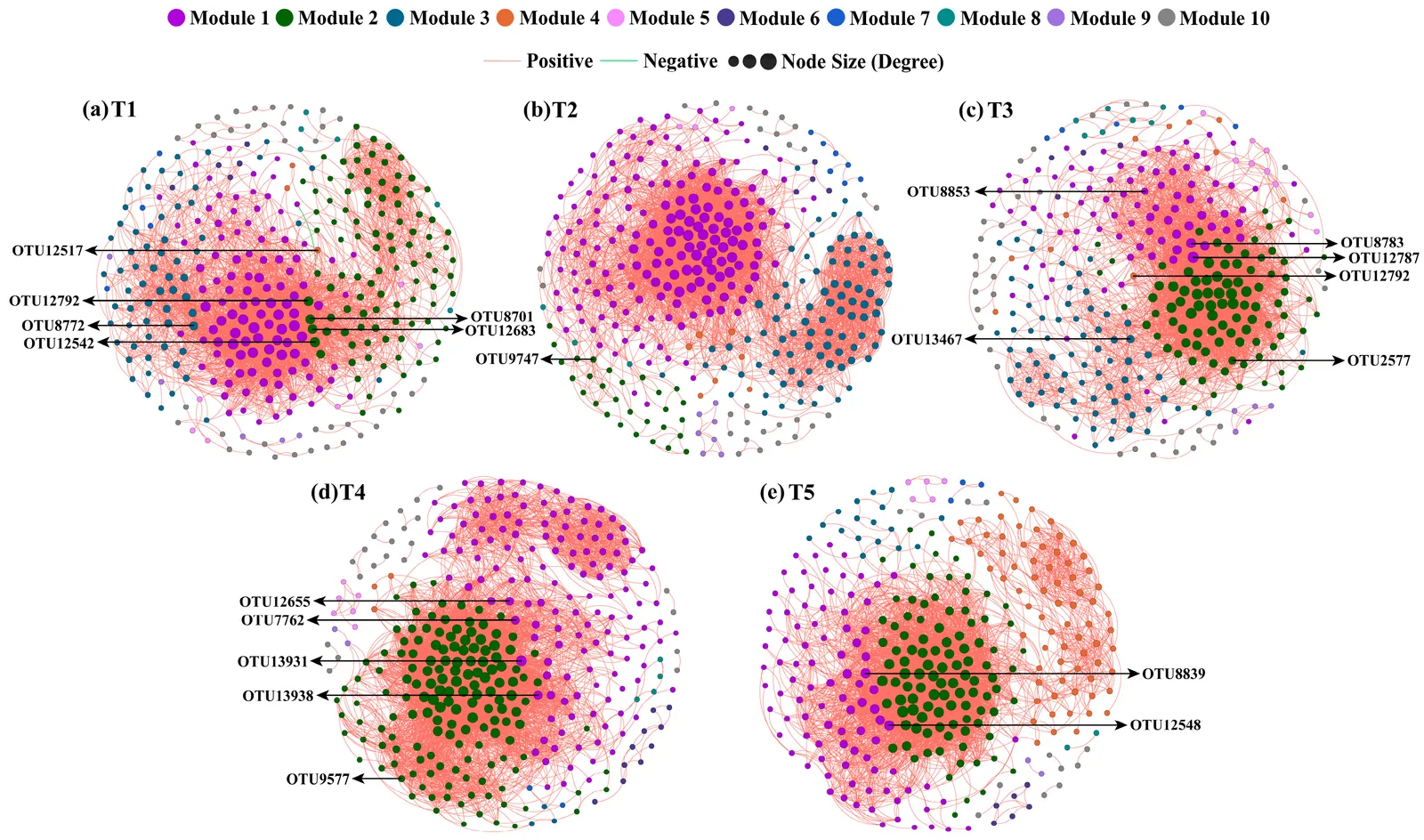
Co-occurrence network of straw-decomposing bacteria under different nitrogen source treatments. (**a**) T1—no N fertilizer addition; (**b**) T2—urea; (**c**) T3—calcium nitrate; (**d**) T4—yeast derivatives; (**e**) T5—glutamate.

**Figure 9:**
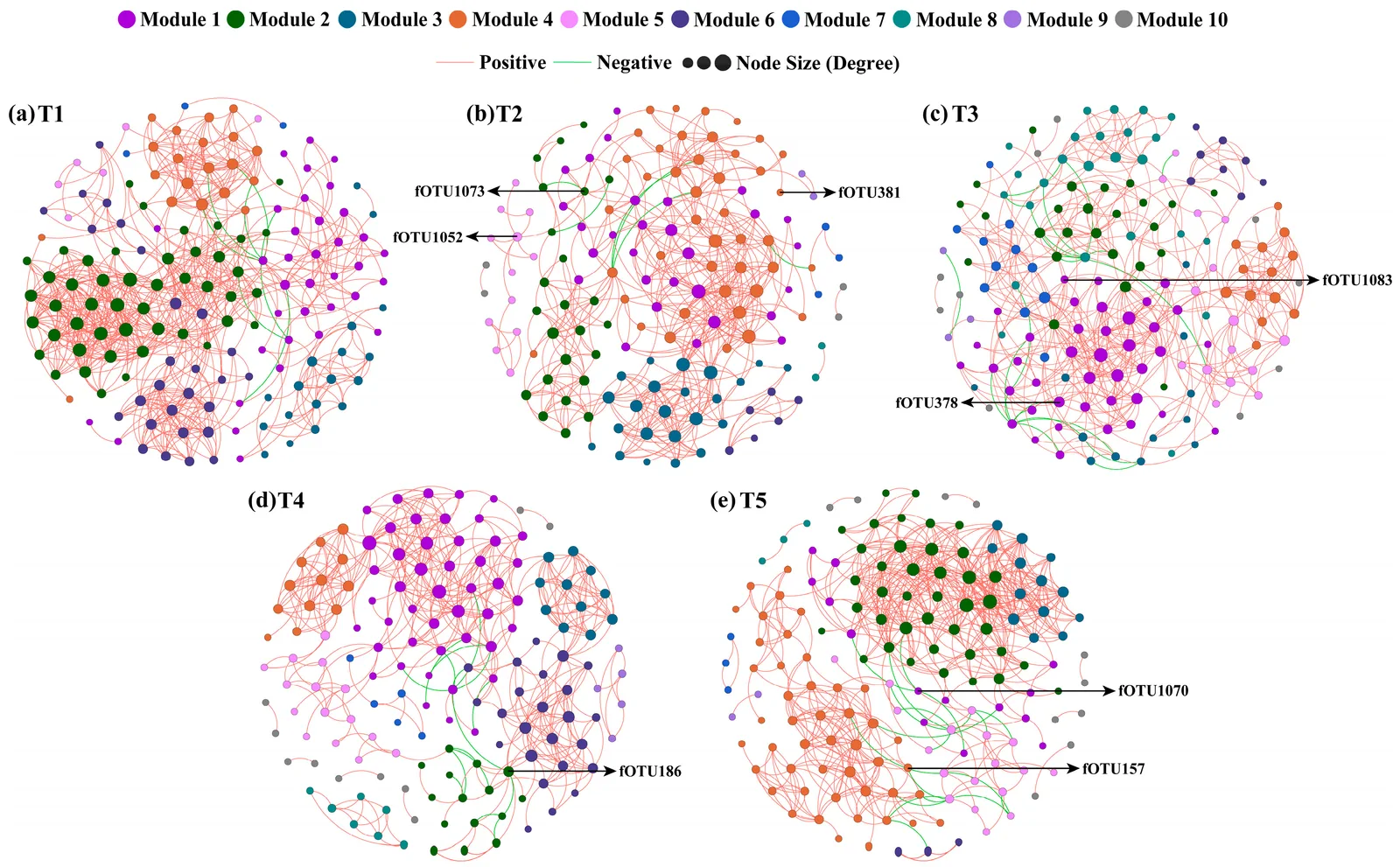
Co-occurrence network of straw-decomposing fungi under different nitrogen source treatments. (**a**) T1—no N fertilizer addition; (**b**) T2—urea; (**c**) T3—calcium nitrate; (**d**) T4—yeast derivatives; (**e**) T5—glutamate.

The residue-decomposing bacterial communities under T4 treatment exhibited a closely connected co-occurrence network with complex interaction characterized by the highest edge density, average degree, and betweenness centralization and the lowest average path length (Figure 8 and Table S2). In contrast, the co-occurrence network of residue-decomposing fungi exhibited elevated edge density, average degree, and degree centralization and reduced average path length under the T1 and T5 treatments (Figure 9 and Table S3). A subset of OTUs in the bacterial network, belonging to *Chitinophaga*, *Chthoniobacter*, *Tundrisphaera*, and *Altererythrobacter*, served as connectors, while additional OTUs such as *Luteolibacter*, *Olivibacter*, *Emticicia*, *Sphingomonas*, *Candidatus_Xiphinematobacter*, and *Devosia* were identified as module hubs, playing pivotal roles within the module (Table 2). In the fungal co-occurrence network, *Vishniacozyma*, *Meyerozyma*, *Candida*, *Pseudogymnoascus*, *Exophiala*, *Alternaria*, and *Schizothecium* emerged as key nodes (Table 3). Furthermore, the complexity of the bacterial–fungal interaction network intensified with prolonged decomposition, exhibiting peak edge density, average degree, clustering coefficient, degree centralization, and reduced average path length but diminished modularity at D180 (Figure S6 and Table S4). Notably, a higher number of key nodes were identified during this phase, with a notable proportion of bacterial OTUs such as *Brevundimonas*, *Mesorhizobium*, *Asticcacaulis*, and *TM7a* (Figure S6d).

We further used a random forest model to quantify the contribution importance of different microbial indicators to straw decomposition rate, aiming to clarify the association between community characteristics and decomposition performance (Figure S7a,b). The results showed that the *R*^2^ values of both models were above 0.8, indicating a good fit of the models. Meanwhile, across the two organic N treatments, microbial alpha-/beta-diversity and identified key nodes of microbial networks have varying degrees of impact on straw decomposition.

## 4. Discussion

Despite the significance of straw return practice in advancing agricultural sustainability, the decomposition of straw is typically slow under natural conditions [40], resulting in delayed nutrient release and limiting the potential benefits of C sequestration and yield enhancement. Our investigation, utilizing straw mesh bag burial experiments, revealed that soil type shaped the ecological preferences of straw-decomposing microbial communities, thereby influencing the rate of straw decomposition. Additionally, the incorporation of organic N sources was found to enhance microbial interactions involved in straw decomposition, consequently accelerating nutrient release. This finding suggests a promising N optimization strategy for future straw utilization practices.

### 4.1. Soil Properties Drove Divergent Straw Decomposition Dynamics by Shaping Microbial Ecological Preference

A cross-climate soil transplantation experiment confirmed that climate was crucial for the regulation of straw decomposition and the involved microbial community structure [41]. Zhao et al. [42] found that straw decomposition was more effective in highly fertile soils, attributed to the notable rise in soil bacterial and fungal biomass. In this study, lime concretion black soil exhibited the highest rates of straw decomposition and C release compared to the other tested soils (Figure 1 and Figure S1), possibly due to the favorable conditions provided by the higher temperature and moisture levels at the beginning of residue decomposition [43]. Moreover, the mildly acidic conditions might facilitate colonization of fungal species [44], such as Leotiomycetes, which can enhance nutrient acquisition in acidic soil environments and was notably enriched in this soil (Figure S5b). The recognized capacity of fungi to break down recalcitrant compounds like lignin may expedite the decomposition of straw [45]. However, the decomposition rate of maize residue was lower in yellow-cinnamon soil (Figure 1), which is likely related to the microbial ecological strategies influenced by its enriched C and N environment. Eutrophic conditions heightened resource competition among microbial populations, leading them to prioritize energy and resources towards maximizing their growth rates over decomposing recalcitrant substrates [46]. Additionally, fluvo-aquic soil is characterized by its high sand content, limited soil water retention capacity, and low nutrient availability. The restricted microbial metabolic activity largely led to a reduction in the degradation and utilization of straw components (Figure 1) [47,48].

Deterministic and stochastic processes jointly drive the construction of microbial communities, which is crucial for maintaining ecosystem functions [49,50,51,52]. Deterministic processes are mainly manifested as ecological selection, where abiotic factors (such as environmental conditions) and biological factors (such as inter-species interactions) shape distinct niches that influence community assembly [53]. Conversely, stochastic processes, which involve random births/deaths and dispersal events, can greatly affect the microbial distribution and result in species composition patterns that are indistinguishable from those arising randomly [54]. Consistent with the findings of Sun et al. [55], our research found the straw-decomposing bacterial community construction was initially and greatly affected by stochastic processes (Figure 5a). Bacteria benefit from their rapid proliferation and preference for abundant and relatively homogeneous substrates during the initial decomposition of labile C components, such as hemicellulose [47]. The copiotrophic organisms *Pseudomonas* and *Pantoea*, which are especially capable of quickly responding to fresh straw inputs [56,57,58], showed significantly higher abundance on D14 of straw decomposition (Figure 6). As degradation advanced, a greater proportion of recalcitrant components, e.g., lignin, decomposed and more microbial metabolites continuously accumulated, creating a more complex microhabitat that accommodated higher species diversity (Figure 3a,b). The assembly of bacterial communities has gradually shifted from being dominated by stochastic processes to being dominated by deterministic processes (especially heterogeneous selection) (Figure 5a). A pronounced geographic distance decay effect on the community structure of straw decomposers was observed, particularly evident at D180 (Figure S4). Meanwhile, Gram-positive bacteria *Bacillus* and *Curtobacterium*, which can efficiently participate in the degradation of straw recalcitrant components [59,60], exhibit strong adaptation to fluvo-aquic soil with increased relative abundance in the late decomposition stage (Figure 6). The genera *Luteibacter* and *Chitinophaga* have been identified as participating in the decomposition of straw lignocellulose via cellulase systems [61,62] and may exhibit a competitive advantage in lime concretion black soil (Figure 6). This observation implies that environmental variables shaped the community structure through strong screening in the later stage of straw decomposition, leading to the differentiation of specific bacterial groups with adaptability to different environments [63].

Different from the pattern of bacterial communities, straw-decomposing fungal community assembly was primarily governed by stochastic processes during the entire decomposition process and dispersal limitation was particularly predominant at D180 (Figure 5c). Meanwhile, a strong correlation between fungal community structure and environmental variables was also observed during this period (Figure 4d), which may reflect the potential constraints of the environment on efficient recalcitrant substance decomposers. However, the specific decomposer composition depended more on random events (such as random spore settling), spatial isolation, or dispersal ability [64], rather than being strictly screened by environmental conditions as in bacteria. The straw-decomposing fungal community composition also shifted from genera such as *Alternaria*, *Rhizopus*, *Cladosporium*, and *Meyerozyma* to those with potentially stronger degradation ability, such as *Chaetomium*, *Schizothecium*, and *Apodus* (Figure 7).

Furthermore, the dynamics of the interaction network between straw-decomposing bacteria and fungi suggest that microbial communities enhance their connectivity and cooperation to optimize resource utilization efficiency in response to the challenges posed by limited and intricate resources in the later stages (Figure S6 and Table S4). While fungi have been traditionally recognized for their role in decomposing recalcitrant compounds, recent research has highlighted the importance of bacterial activity in degrading cellulose and lignin [61,65]. The decomposing bacterial–fungal network analysis revealed a notable increase in the number of key nodes within the network at D180 (Figure S6). For example, bacteria such as *Brevundimonas*, *Mesorizobium*, *Asticcacaulis*, and *TM7a*, which showed relatively high abundance, were identified as module hubs (Figure S6). Brevundimonas is known for its cellulase secretion capabilities and its ability to thrive in low-nutrient environments [66,67]. *Asticcacaulis* has demonstrated significant efficiency in breaking down cellulose substrates [68] and *TM7a* has been linked to the decomposition of ryegrass litter [69]. These key taxa likely play a crucial role in maintaining the functional resilience of communities in complex heterogeneous environments.

### 4.2. Organic Nitrogen Sources Facilitated Straw Decomposition Through Enhanced Microbial Interactions

Straw addition without external N input led to strong N fixation by microorganisms to meet their growth requirements [70], as evidenced by the notable N accumulation associated with the residues at D180 in this study (Figure S2). The addition of exogenous N can mitigate microbial N limitation under high-C/N-ratio straw returning, consequently expediting the release of C and N nutrients from straw residues [71,72]. However, when crop residues with a low C/N ratio are incorporated into the soil, they can provide sufficient N for microbial decomposition and crop growth, thus minimizing or obviating the need for supplemental N fertilizers and reducing production costs. As reported by Song et al. [73], the incorporation of legume straw provides comprehensive benefits superior to those of gramineous crop residues in terms of soil quality improvement and carbon footprint reduction. Furthermore, N application appeared to shape more similar straw-decomposing bacterial communities (Figure S4), with the copiotrophic attribute phyla Proteobacteria and Bacteroidota being the dominant bacterial phyla in different soils (Figure S5). On the contrary, straw-decomposing fungal community structure was sensitive to the incorporation of different N sources (Figure 4b). Moreover, organic N sources (yeast derivatives and glutamate) were more efficient in enhancing straw decomposition and nutrient release compared to inorganic N sources (Figure 1 and Figures S1 and S2). This could be partly attributed to the multifaceted support provided by organic N sources, which not only supply N but also serve as energy and C sources to microorganisms, thereby facilitating a more holistic nutritional framework that sustains effective straw decomposition processes [74].

Based on the random forest model, we further quantitatively evaluated the microbial contribution to straw decomposition under organic N addition. The decomposition process was not governed by a single factor; community structure, diversity, and key taxa were strongly associated with decomposition performance, and their relative importance shifted to some extent due to the N source type. This indicates that microbially mediated straw decomposition exhibits a certain degree of nitrogen source dependence. It is worth noting that the impact of organic N sources on straw decomposition displayed distinct temporal patterns and the addition of yeast derivatives demonstrated notable advantages during the initial decomposition phase by enhancing the extensive interactions among bacterial decomposers (Figure 1, Figure 8d and Figures S1 and S2). The readily available sugars, amino acids, and small-molecule peptides derived from yeast derivatives are preferentially taken up by bacterial decomposers, which substantially lowers the metabolic energy cost for microorganisms to obtain C substrates, accelerates bacterial growth, and fosters the establishment of a highly efficient decomposition network, thereby supporting early rapid decomposition [75,76]. The key taxa *Altererythrobacter*, *Sphingomonas*, *Candidatus_Xiphinematobacter*, *Devosia*, and *Chitinophaga* potentially sustained the straw-decomposing microbial co-occurrence network under the yeast derivative treatment (Table 2). *Altererythrobacter* is closely associated with C conversion and the activity of various decomposition enzymes, playing a crucial role in residue decomposition [77]. *Sphingomonas* can mitigate the inhibition of C decomposition metabolites through diverse xylose metabolism pathways and enable the simultaneous utilization of glucose and xylose from straw for extracellular polysaccharide production, thereby enhancing straw nutrient utilization [78]. *Candidatus_Xiphinematobacter* is considered a specific symbiotic bacterium capable of utilizing host-secreted specific C sources [79], with its growth affected by changes in pH and temperature [80]. Owing to its adaptable and versatile genome, *Devosia* can thrive in diverse environments through various short-peptide transport systems and evolutionary advances in key genes, suggesting significant potential in organic pollutant degradation and cellulose breakdown [81,82].

On the contrary, the promotion of glutamate addition on straw decomposition became apparent in the later decomposition stage (Figure 1 and Figures S1 and S2). This delayed response may be attributed to the preferential utilization of readily decomposable substrates by microorganisms under conditions of sufficient C availability, which may weaken the early utilization of glutamate [83]. As labile C becomes depleted, the synthesis of permeases is progressively induced [70], facilitating the efficient uptake of glutamate. Once internalized, glutamate is converted through deamination to generate α-ketoglutarate, which subsequently serves as an energy source for fungal decomposers [84]. This process supports the development of intricate fungal interaction networks in the later decomposition phase (Figure 9e). Meanwhile, α-ketoglutarate, acting as a cofactor for histone demethylase, can enhance the expression of genes encoding lignin-degrading enzymes by modulating chromatin structure [85], therefore achieving a significant acceleration of straw decomposition by glutamate. Genera *Alternaria* and *Schizothecium* largely contributed to this result (Table 3), which is widely present in nature, with many species known to be plant pathogens [86,87]. These key microbial strains may provide an important reference for future exploration of N optimization measures for straw return and the development of functional microbial agents.

## 5. Conclusions

This study demonstrated that the assembly of bacterial and fungal communities involved in straw decomposition exhibited a clear succession in community composition throughout the decomposition process, and was dominated by deterministic and stochastic processes, respectively. Different bacterial and fungal communities displayed closer interactions at the late stages of straw decomposition. N fertilization, particularly organic N sources, accelerated the decomposition process. In addition, yeast derivatives notably enhanced straw decomposition and nutrient release during the initial stages by promoting interactions among straw-decomposing bacterial communities, especially in fluvo-aquic soil, lime concretion black soil, and cinnamon soil. *Altererythrobacter*, *Sphingomonas*, *Candidatus_Xiphinematobacter*, *Devosia*, and *Chitinophaga* potentially played crucial roles in the yeast derivative-incorporated soil. Furthermore, glutamate mainly facilitated the late stages of decomposition by enhancing the interactions among straw-decomposing fungal members, with *Alternaria* and *Schizothecium* identified as key nodes in the decomposition of crop residues. These findings offer insights into N optimization for the efficient utilization of straw nutrients, which potentially contributed to the global enhancement of SOC stocks. Future investigations should focus on isolating and evaluating the functional microbial strains under various soil conditions and exploring combined optimized N management approaches to improve decomposition efficiency.

## Figures and Tables

**Table 1:** Detailed input amounts for each nitrogen source treatment.

Treatment	N Source	Straw Mass (g)	N Source Mass (g)	C Content of N Source (%)	N Content of N Source (%)	Total C (g)	Total N (g)	C/N
T1	No N addition	14	0	—	—	5.9238	0.0637	93
T2	Urea	14	0.378	19.98	46.62	5.9993	0.2399	25
T3	Calcium nitrate	14	1.015	0	17.06	5.9238	0.2369	25
T4	Yeast derivatives	14	1.7703	41.1	11.43	6.6514	0.2660	25
T5	Glutamate	14	2.1966	40.78	9.52	6.8196	0.2728	25

**Table 2:** Key node information of straw-decomposing bacterial co-occurrence network.

Treatment	OTU ID	Node Type	Relative Abundance (%)	Phylum	Family	Genus
T1	OTU12517	Connectors	0.1477	Bacteroidota	Chitinophagaceae	*Chitinophaga*
	OTU12542	Module hubs	0.2918	Proteobacteria	Micropepsaceae	*norank_f__Micropepsaceae*
	OTU12683	Module hubs	0.0223	Proteobacteria	Moraxellaceae	*norank_f__Moraxellaceae*
	OTU12792	Module hubs	0.0182	Bacteroidota	Chitinophagaceae	*norank_f__Chitinophagaceae*
	OTU8701	Module hubs	0.0115	Verrucomicrobiota	Rubritaleaceae	*Luteolibacter*
	OTU8772	Module hubs	0.0352	Actinobacteriota	Ilumatobacteraceae	*norank_f__Ilumatobacteraceae*
T2	OTU9747	Module hubs	0.0691	Bacteroidota	Sphingobacteriaceae	*Olivibacter*
T3	OTU8853	Connectors	0.0350	Verrucomicrobiota	Chthoniobacteraceae	*Chthoniobacter*
	OTU2577	Connectors	0.0181	Planctomycetota	Isosphaeraceae	*Tundrisphaera*
	OTU8783	Module hubs	0.0351	Verrucomicrobiota	Rubritaleaceae	*Luteolibacter*
	OTU12787	Module hubs	0.0226	Verrucomicrobiota	Chthoniobacteraceae	*Chthoniobacter*
	OTU13467	Module hubs	0.0261	Bacteroidota	Spirosomaceae	*Emticicia*
	OTU12792	Module hubs	0.0125	Bacteroidota	Chitinophagaceae	*norank_f__Chitinophagaceae*
T4	OTU9577	Connectors	0.0455	Proteobacteria	Sphingomonadaceae	*Altererythrobacter*
	OTU7762	Module hubs	0.0300	Proteobacteria	Sphingomonadaceae	*Sphingomonas*
	OTU12655	Module hubs	0.0226	Verrucomicrobiota	Xiphinematobacteraceae	*Candidatus_Xiphinematobacter*
	OTU13938	Module hubs	0.0166	Proteobacteria	Devosiaceae	*Devosia*
	OTU13931	Module hubs	0.0121	Bacteroidota	Chitinophagaceae	*Chitinophaga*
T5	OTU8839	Module hubs	0.0576	Proteobacteria	Devosiaceae	*unclassified_f__Devosiaceae*
	OTU12548	Module hubs	0.0198	Actinobacteriota	67-14	*norank_f__67-14*

**Table 3:** Key node information of straw-decomposing fungal co-occurrence network.

Treatment	OTU ID	Node Type	Relative Abundance (%)	Phylum	Family	Genus
T2	fOTU381	Connectors	0.0229	Basidiomycota	Bulleribasidiaceae	*Vishniacozyma*
	fOTU1073	Connectors	3.4510	Ascomycota	Debaryomycetaceae	*Meyerozyma*
	fOTU1052	Module hubs	0.0423	Ascomycota	Saccharomycetales_fam_Incertae_sedis	*Candida*
T3	fOTU1083	Connectors	8.1270	Ascomycota	Pseudeurotiaceae	*Pseudogymnoascus*
	fOTU378	Connectors	0.2111	Ascomycota	Herpotrichiellaceae	*Exophiala*
T4	fOTU186	Module hubs	5.4324	Ascomycota	Chaetomiaceae	*unclassified_f__Chaetomiaceae*
T5	fOTU1070	Connectors	8.2013	Ascomycota	Pleosporaceae	*Alternaria*
	fOTU157	Connectors	0.2828	Ascomycota	Lasiosphaeriaceae	*Schizothecium*

## Data Availability

The data presented in this study are available on request from the corresponding author.

## Supplementary Materials

The following supporting information can be downloaded at https://www.mdpi.com/article/10.3390/microorganisms14081753/s1, Table S1: Climate conditions and soil basic physicochemical properties of the experiment sites. Table S2: Topological structure of straw-decomposing bacterial co-occurrence network. Table S3: Topological structure of straw-decomposing fungal co-occurrence network. Table S4: Topological structure of bacterial–fungal co-occurrence network; Figure S1: Straw carbon release under various nitrogen source treatments in different soil types. *Y*—straw C and N residual rate (%); t—decomposition time (yr); e—natural constant; *R*^2^—the goodness-of-fit of the equation. Figure S2: Straw nitrogen release under various nitrogen source treatments in different soil types. *Y*—straw C and N residual rate (%); t—decomposition time (yr); *R*^2^—the goodness-of-fit of the equation. Figure S3: Straw cellulose (a), hemicellulose (b), and lignin (c) decomposition rate under various nitrogen source treatments in different soil types. Figure S4: Distance decay relationship (DDR) analysis of straw-decomposing bacterial (a,b) and fungal (c,d) communities. *** *p* < 0.001. Figure S5: The community composition of straw-decomposing bacteria at the phylum level (a) and fungi at the class level (b). Figure S6: Straw-decomposing bacterial–fungal interaction network and key node information at different decomposition times. Circles represent bacterial OTUs, and triangles represent fungal OTUs. Figure S7: Random forest models under (a) yeast derivative (T4) and (b) glutamate (T5) treatment. F_ represents fungal indicators; B_ represents bacterial indicators; PC1 and PC2 are the first two axes of PCoA analysis based on Bray–Curtis distance; and the OTU numbers correspond to key nodes in the co-occurrence network of microorganisms under two different treatments. * *p* < 0.05, ** *p* < 0.01.
