# Supplementary material for: Nitrogen Sources Reshape Microbial Community Assembly and Interaction Networks to Regulate Straw Decomposition and Carbon Release in Cropland Soils of the North China Plain
Source: Microorganisms. 2026 Aug 9;14(8):1753. doi: 10.3390/microorganisms14081753 (PMC13515101; doi:10.3390/microorganisms14081753)
Supplement: Supplementary file 1 [file microorganisms-14-01753-s001.zip › microorganisms-4458808-supplementary.pdf]

Table S1. Climate conditions and soil basic physicochemical properties of the experiment sites

Soil type	International classification (FAO-UNESCO)	Site	Geographic location	MAP (mm)	MAT (°C)	pH	SOC (g/kg)	TN (g/kg)	AP (mg/kg)	AK (mg/kg)
Fluvo-aquic soil	Fluvisols	Weihui	35°29′51″N, 114°11′11″E	735.9	15.3	7.26	6.61	1.27	24.21	151.33
		Xinxiang	35°11′16″N, 113°56′56″E	570.4	15.4	8.33	3.61	0.63	6.44	74.00
		Yuanyang	34°47′45″N, 113°40′18″E	488.3	15.0	8.10	6.80	0.73	15.49	166.67
		Shangqiu	34°19′7″N, 115°27′51″E	684.5	15.8	7.50	10.75	0.98	23.39	119.80
Lime concretion black soil	Vertisols	Wuyang	33°36′53″N, 113°38′59″E	1169.5	15.7	5.71	7.88	0.67	16.26	203.33
		Xiping	33°25′16″N, 113°57′25″E	765.1	16.4	5.12	9.14	0.90	20.83	127.50
		Sheqi	33°0′16″N, 112°53′14″E	628.4	16.3	4.96	9.35	1.55	72.79	255.00
Cinnamon soil	Cambisols	Jiaozuo	35°4′2″N, 113°12′8″E	630.6	15.7	7.15	3.93	0.87	12.15	151.33
		Yuzhou	34°16′13″N, 113°20′26″E	459.1	15.7	7.51	7.03	1.03	20.90	118.25
Yellow-cinnamon soil	Luvisols	Wuyang	33°36′25″N, 113°38′58″E	1169.5	15.7	6.33	6.26	0.53	13.01	185.61
		Luohe	33°38′9″N, 114°14′13″E	549.4	15.8	5.27	12.19	1.93	53.40	344.67

Notes: MAP—mean annual precipitation; MAT—mean annual temperature; SOC—soil organic carbon; TN—total nitrogen; AP—available phosphorus; AK—available potassium.

Table S2. Topological structure of straw-decomposing bacterial co-occurrence network

Topological Parameters	T1	T2	T3	T4	T5
Number of edges	3634	4927	3815	6350	4401
Number of nodes	340	355	331	347	317
Edge density	0.06	0.08	0.07	0.11	0.09
Average degree	21.38	27.76	23.05	36.6	27.77
Average path length	3.26	3.45	3.32	3	3.37
Diameter	9	13	12	10	12
Clustering coefficient	0.56	0.66	0.61	0.66	0.65
Betweenness centralization	0.04	0.06	0.07	0.08	0.07
Degree centralization	0.19	0.19	0.22	0.22	0.23

Notes: T1—no N fertilizer addition; T2—urea; T3—calcium nitrate; T4—yeast derivatives; T5—glutamate.

Table S3. Topological structure of straw-decomposing fungal co-occurrence network

Topological Parameters	T1	T2	T3	T4	T5
Number of edges	726	474	530	517	656
Number of nodes	157	149	162	150	152
Edge density	0.06	0.04	0.04	0.05	0.06
Average degree	9.25	6.36	6.54	6.89	8.63
Average path length	4.2	4.31	4.37	4.29	3.76
Diameter	10	13	11	11	9
Clustering coefficient	0.62	0.47	0.49	0.56	0.55
Betweenness centralization	0.14	0.12	0.17	0.13	0.14
Degree centralization	0.1	0.09	0.09	0.09	0.14

Notes: T1—no N fertilizer addition; T2—urea; T3—calcium nitrate; T4—yeast derivatives; T5—glutamate.

Table S4. Topological structure of bacterial–fungal co-occurrence network

Topological Parameters	D14	D90	D180
Number of edges	865	1374	28958
Number of nodes	205	279	795
Edge density	0.04	0.04	0.09
Average degree	8.44	9.85	72.85
Average path length	4.34	3.59	2.84
Diameter	12	8	10
Clustering coefficient	0.56	0.47	0.63
Betweenness centralization	0.14	0.06	0.02
Degree centralization	0.2	0.16	0.27

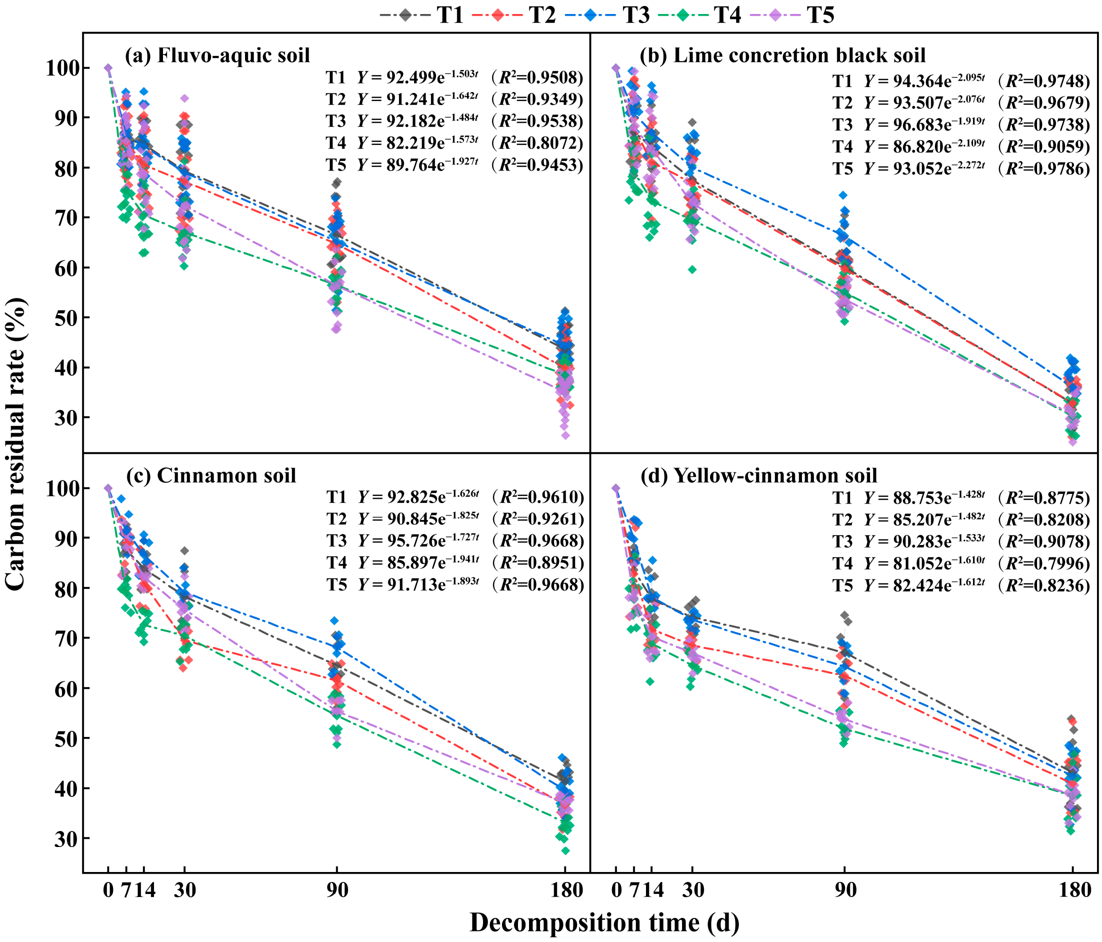

Figure S1. Straw carbon release under various nitrogen source treatments in different soil types. *Y*—straw C and N residual rate (%); t—decomposition time (yr); e—natural constant; R^2—the goodness-of-fitting of the equation.

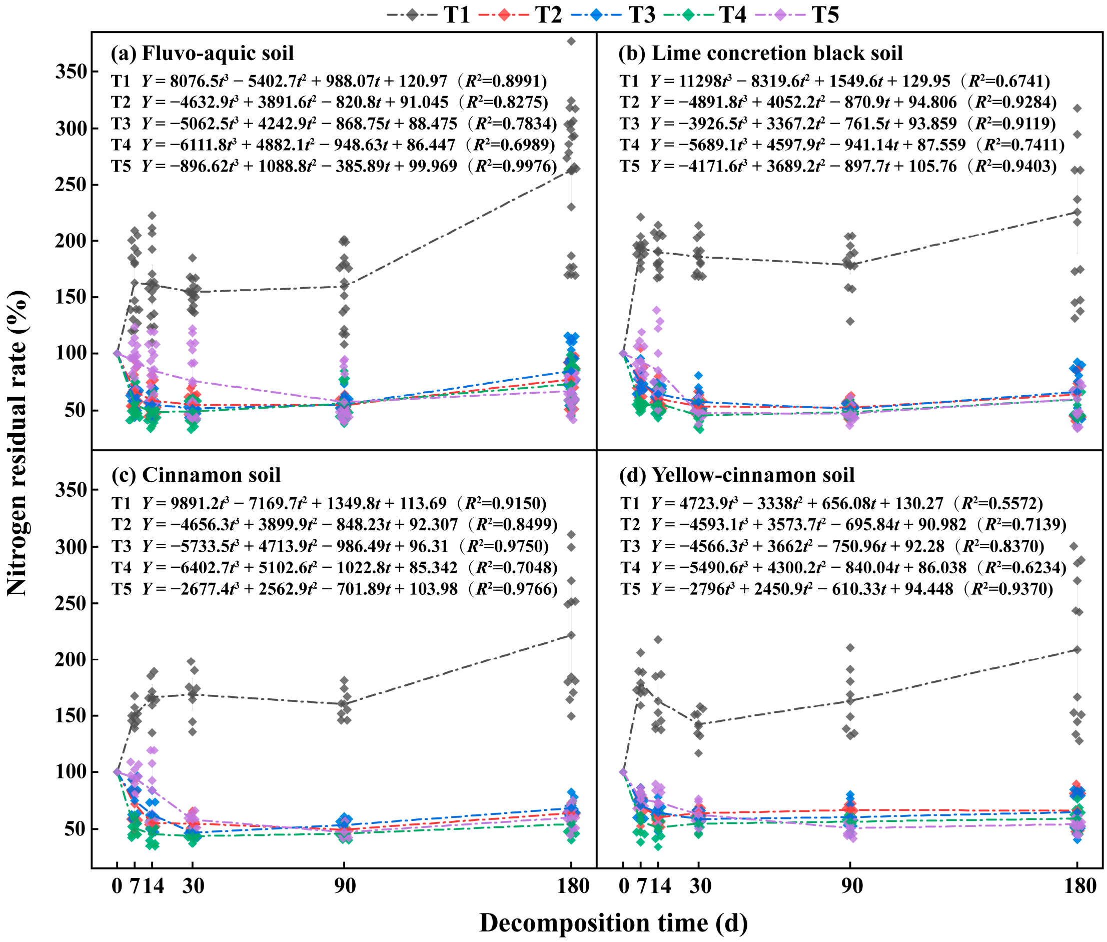

Figure S2. Straw nitrogen release under various nitrogen source treatments in different soil types. Y—straw C and N residual rate (%); t—decomposition time (yr); R^2—the goodness-of-fitting of the equation.

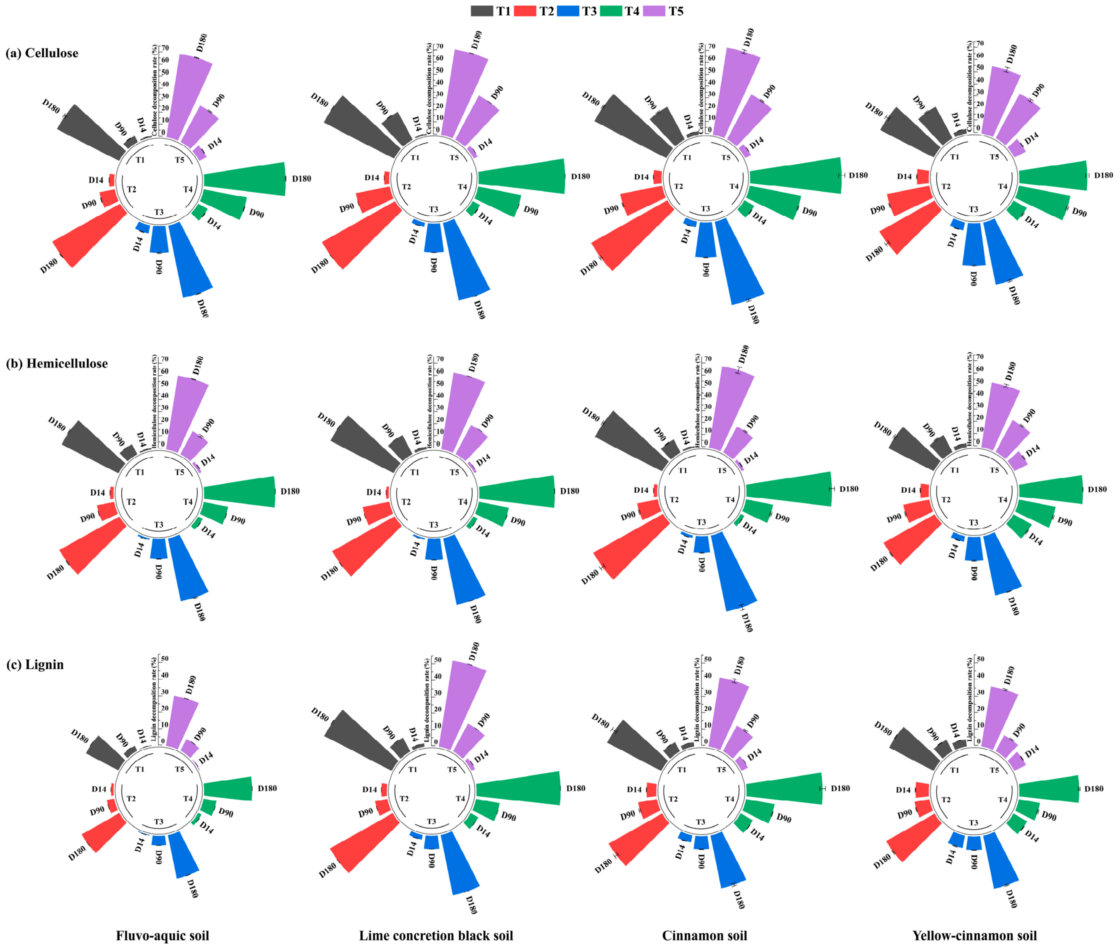

Figure S3. Straw cellulose (a), hemicellulose (b), and lignin (c) decomposition rate under various nitrogen source treatments in different soil types.

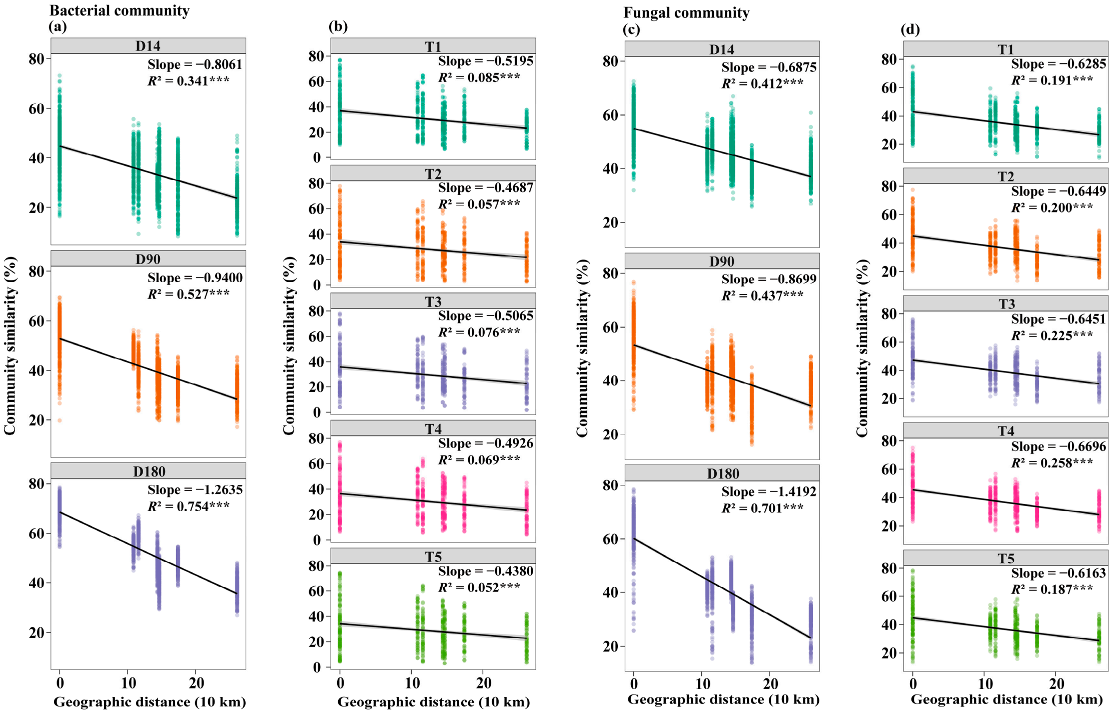

Figure S4. Distance decay relationship (DDR) analysis of straw-decomposing bacterial (a,b) and fungal (c,d) communities. *** $p < 0.001$.

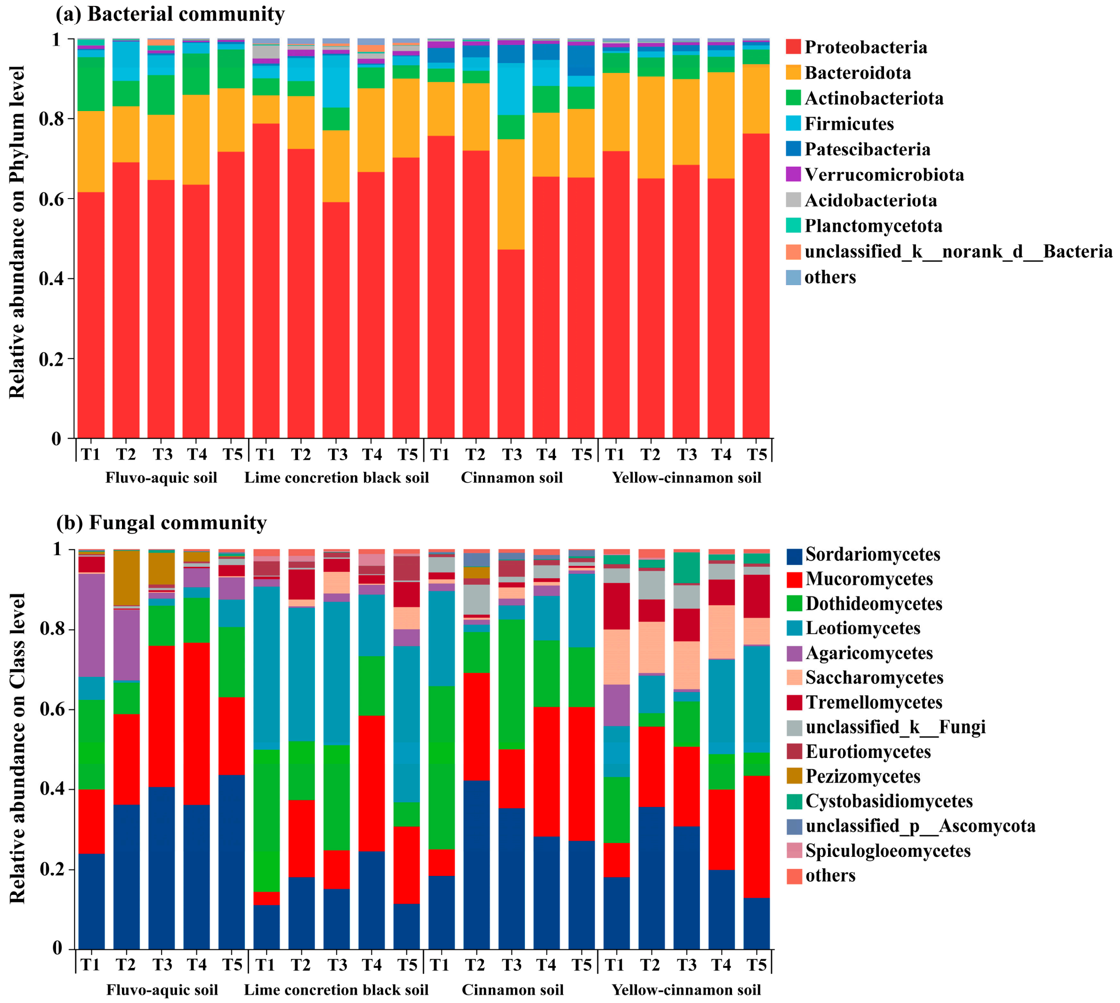

Figure S5. The community composition of straw-decomposing bacteria at the phylum level (a) and fungi at the class level (b).

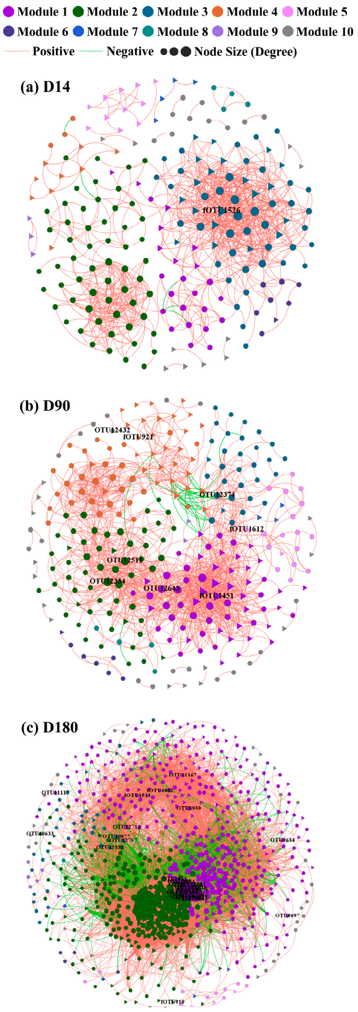

(d)

Time	OTU ID	Node type	Relative abundance (%)	Phylum	Genus
D14	fOTU1526	Module hubs	0.8312	Ascomycota	*Wickerhamomyces*
D90	fOTU1612	Connectors	0.0167	Ascomycota	*Chaetomium*
	fOTU921	Connectors	0.7740	Ascomycota	*Cheilymenia*
	OTU12643	Connectors	0.0429	Proteobacteria	*Sphingobium*
	OTU12432	Connectors	0.0256	Proteobacteria	*unclassified_f__Oxalobacteraceae*
	OTU12374	Module hubs	0.0825	Proteobacteria	*Rhodopseudomonas*
	OTU12511	Module hubs	0.1423	Bacteroidota	*Mucilaginibacter*
	OTU12354	Module hubs	0.0608	Proteobacteria	*Sphingopyxis*
	fOTU1451	Module hubs	0.0100	Ascomycota	*unclassified_o__Sordariales*
D180	OTU12711	Connectors	0.1485	Bacteroidota	*Flavobacterium*
	OTU9634	Connectors	0.1057	Actinobacteriota	*Nocardioides*
	OTU12519	Connectors	0.0248	Bacteroidota	*Mucilaginibacter*
	OTU8959	Connectors	0.0201	Bacteroidota	*norank_f__Microscillaceae*
	OTU11167	Connectors	0.0140	Actinobacteriota	*Glycomyces*
	OTU11117	Connectors	0.0115	Proteobacteria	*unclassified_c__Alphaproteobacteria*
	OTU9697	Connectors	0.0107	Planctomycetota	*Singulisphaera*
	fOTU1082	Connectors	2.5347	Mucoromycota	*Rhizopus*
	fOTU1513	Connectors	0.0570	Ascomycota	*Hirsutella*
	OTU10633	Connectors	0.0132	Proteobacteria	*unclassified_c__Alphaproteobacteria*
	fOTU910	Connectors	0.0405	Ascomycota	*Trichocladium*
	OTU8943	Module hubs	0.5807	Actinobacteriota	*unclassified_f__Microbacteriaceae*
	OTU8941	Module hubs	0.5462	Proteobacteria	*Brevundimonas*
	OTU12323	Module hubs	0.4505	Proteobacteria	*Mesorhizobium*
	OTU12396	Module hubs	0.2407	Proteobacteria	*Asticcacaulis*
	OTU12609	Module hubs	0.0987	Proteobacteria	*norank_f__Diplorickettsiaceae*
	OTU12790	Module hubs	0.0948	Proteobacteria	*Rhizobacter*
	OTU12314	Module hubs	0.0790	Verrucomicrobiota	*Terrimicrobium*
	OTU12552	Module hubs	0.0711	Proteobacteria	*Sphingoaurantiacus*
	OTU12634	Module hubs	0.0684	Bacteroidota	*Mucilaginibacter*
	OTU12560	Module hubs	0.0651	Myxococcota	*norank_f__BIrii41*
	OTU12483	Module hubs	0.0541	Bacteroidota	*Ferruginibacter*
	OTU12439	Module hubs	0.0355	Armatimonadota	*norank_f__Fimbriimonadaceae*
	OTU12716	Module hubs	0.0289	Proteobacteria	*norank_f__norank_o__Rhodospirillales*
	OTU12541	Module hubs	0.0286	Bdellovibrionota	*Bdellovibrio*
	OTU13371	Module hubs	0.0151	Bdellovibrionota	*Bdellovibrio*
	fOTU335	Module hubs	1.1018	Basidiomycota	*unclassified_o__Spiculogloeales*
	fOTU365	Module hubs	0.0969	Ascomycota	*Knufia*
	OTU12322	Module hubs	0.5940	Patescibacteria	*TM7a*
	OTU10077	Module hubs	0.0336	Bacteroidota	*Pedobacter*
	OTU12757	Module hubs	0.0309	Bacteroidota	*Edaphobaculum*

Figure S6. Straw-decomposing bacterial–fungal interaction network and key node information at different decomposition times. Circles represent bacterial OTUs, and triangles represent fungal OTUs.

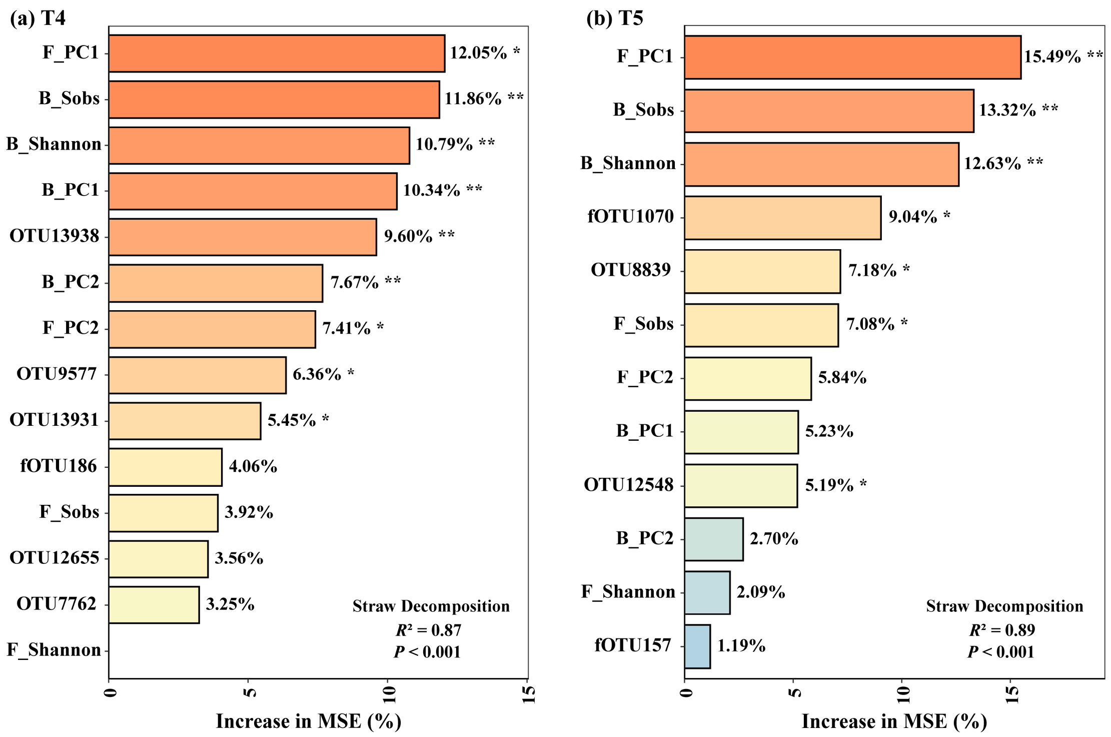

Figure S7. Random forest models under (a) yeast derivatives (T4) and (b) glutamate (T5) treatment. F_ represents fungal indicators, B_ represents bacterial indicators; PC1 and PC2 are the first two axes of PCoA analysis based on Bray-Curtis distance; the OTU numbers correspond to key nodes in the co-occurrence network of microorganisms under two different treatments. * $p < 0.05$, ** $p < 0.01$.
